# A Rapid Review of the Impact of Family-Based Digital Interventions for Obesity Prevention and Treatment on Obesity-Related Outcomes in Primary School-Aged Children

**DOI:** 10.3390/nu14224837

**Published:** 2022-11-15

**Authors:** Li Kheng Chai, Rebecca Farletti, Leila Fathi, Robyn Littlewood

**Affiliations:** 1 Health and Wellbeing Queensland, Queensland Government, Brisbane, QLD 4064, Australia; 2 School of Human Movement and Nutrition Sciences, The University of Queensland, Brisbane, QLD 4072, Australia

**Keywords:** children, family, obesity, virtual, web-based, program, review

## Abstract

Virtual delivery of obesity prevention and treatment programs may be effective for supporting children and families to adopt healthy lifestyle changes while enhancing program accessibility. This rapid review aimed to summarize the impact of family-based digital interventions for childhood obesity prevention and treatment. Four databases were searched up to February 2021 for trials of interactive digital programs aimed to prevent and/or treat obesity in children aged 5–12 years and reported diet, physical activity, sedentary behavior, sleep, or weight-related outcomes in children. A total of 23 publications (from 18 interventions) were included. Behavior change theories were used in 13 interventions with “Social Cognitive Theory” applied most frequently (*n* = 9). Interventions included websites (*n* = 11), text messaging (*n* = 5), video gaming (*n* = 2), Facebook (*n* = 3), and/or mobile applications (*n* = 2). Studies reported changes in body mass index (BMI; *n* = 11 studies), diet (*n* = 11), physical activity (*n* = 10), screen time (*n* = 6), and/or sleep (*n* = 1). Significant improvements were reported for diet (*n* = 5) or physical activity (*n* = 4). Two of the six interventions were effective in reducing screen time. Digital interventions have shown modest improvements in child BMI and significant effectiveness in diet and physical activity, with emerging evidence supporting the use of social media and video gaming to enhance program delivery.

## 1. Introduction

Childhood obesity is a global public health challenge of the 21st century and has been designated as one of the five key policy priorities of the World Obesity Federation. Evidence suggests that obesity is linked with an increased risk of complications and mortality from coronavirus disease 2019 (COVID-19), which further adds to global health concerns [1]. Moreover, the need for self-isolation and/or quarantine during the COVID-19 pandemic is prompting many to rely on processed food with a longer shelf life (instead of fresh produce). Considering this, the ongoing burden of the pandemic threatens the risk of excessive weight gain. A report published by the Centers for Disease Control and Prevention [2] indicated that the body mass index (BMI) increment rates almost doubled between pre-pandemic and pandemic periods in children aged 2–19 years across all BMI categories, except those who were underweight. These findings highlighted the importance of preventing excess weight gain in children during and following the pandemic, as well as in the events of future public health emergencies, by increasing access to programs that promote healthy behaviors [2].

Conventional family-based obesity interventions are effective in promoting health behavior changes for children. These programs usually engage at least one parent and the child to encourage healthy behavior change by coaching parents in weekly, group-based sessions which focused on goal-setting, problem-solving, monitoring, and role-modelling healthy behaviors. However, the common barriers faced by participants include geographical distance, ease of access, time constraints, and weight-related stigma. Research suggests that web-based programs may be effective in supporting children and families to adopt healthy lifestyle changes while enhancing program accessibility [3]. COVID-19 has led to an increase in using online digital platforms for remote work, education, and healthcare [4]. Globally, there is a commitment to the development and implementation of digital health technologies for the detection, prevention, and treatment of disease, and the promotion of health and wellbeing. These technologies often include computers, websites, mobile applications (apps), short message services (SMS), digital games, telehealth, and wearables/monitoring devices. The potential scalable reach of these technologies provides a unique opportunity for the implementation of population-wide behavioral interventions.

The current literature has broadly investigated the acceptability and efficacy of technology-based interventions to improve clinical outcomes for children and adolescents [5] and its impact to facilitate communication between caregivers and health professionals in the clinical setting [6]. However, limited reviews of the literature to date have investigated the effectiveness of family-based digital interventions across various health outcomes in the context of obesity prevention or treatment for children. Due to the heterogeneity and differing methodologies employed by studies investigating the effectiveness of digital interventions to improve health outcomes [7], as well as the dynamic development and evolvement of digital technologies and their various applications, a rapid review methodology would be more appropriate than a thorough systematic literature review or meta-analysis [8]. Rapid reviews are useful in fields where change is ongoing, such as the development and advancement of digital technologies. This review utilized a simplified methodology based on a systematic literature review process to produce synthesized knowledge in a time-efficient manner [9]. The aim of this rapid review is to summarize the impact of family-based digital interventions for obesity prevention and treatment in children, with a specific focus on diet, physical activity, sedentary behavior, sleep, and weight-related outcomes in primary school-aged children.

## 2. Materials and Methods

### 2.1. Search Strategy

Peer-reviewed journal articles were searched in February 2021 in four electronic databases, including MEDLINE (1946 to present), CINAHL (1981 to present), PsycINFO (1806 to present), and Cochrane Library (including Cochrane Reviews, the Database of Abstracts of Reviews of Effectiveness, the Health Technology Assessment database). Hand-searching of relevant outcome papers of eligible published protocols and the reference lists of relevant systematic reviews were performed to identify other potentially eligible studies. No language or geographical region restrictions were imposed. Unpublished studies or grey literature publications (conference proceedings, blogs, theses, and dissertations) were excluded from this review.

A list of search terms was developed based on previous reviews [10,11], presented in Supplementary Table S1 (see supplementary materials). In brief, the search terms and combinations used were as follows: Family (family OR parent OR carer OR mother OR father) AND child (child OR children OR young person NOT infant) AND digital intervention (online program OR digital tool OR multimedia OR app OR website OR eHealth OR mHealth) AND interactive component (gaming OR music OR animation OR interactive) AND obesity-related outcomes (body mass index OR waist circumference OR nutrition OR physical activity OR sleep).

Web searching was performed in February 2021 to identify digital interventions for obesity prevention and treatment in children, which may not have been indexed or published in the databases searched. The Cochrane Handbook for Systematic Reviews of Interventions [12] recommended that web searching should use a combination of search engines and websites to ensure a wide range of sources are identified and searched in depth. Google Search and Google Scholar were used to search for websites of digital interventions, online programs that were known to the researchers, and associated publications that may be relevant to this review. Due to the basic search interfaces of the search engines, a combination of simplified and natural language key terms, based on the search terms used for searching bibliographic databases (child obesity online prevention program, an online healthy lifestyle program for families), were used. The search was limited to the first 30 hits sorted by relevance.

### 2.2. Inclusion and Exclusion Criteria

#### 2.2.1. Study Design

The review included studies with a parallel control group, including randomized controlled trials (RCTs) and cluster RCTs; quasi-RCTs and cluster quasi-RCTs; controlled before and after studies (CBAs) and cluster CBAs; controlled time series designs. Observational studies, such as longitudinal cohort studies and cross-sectional studies were excluded. Studies could be published in any language or geographic region. Only studies that: (1) compare an online/digital intervention with no intervention or “usual practice” control group, or (2) compare two or more online/digital interventions which aim to improve obesity-related outcomes in children were included. Studies without a comparison arm were excluded.

#### 2.2.2. Participants

Eligible studies involved children who were aged 5–12 years and generally healthy, and/or their parent(s) (i.e., carers, caregivers, guardians). Participants could be grouped as follows: children and parents, whole family, parents only, or child only. Studies that involved children aged below five years, pre-natal or ante-natal, infants, those aged above 12 years (adolescents), or those with chronic health conditions, were excluded.

#### 2.2.3. Interventions

The conditions of interest were interactive digital programs for obesity prevention and treatment interventions in children targeting diet, physical activity, sedentary behavior, sleep, or weight-related measures. Any prevention and/or treatment intervention that aimed to improve obesity-related outcomes in children and was delivered to participants using a digital and/or an online platform as a standalone intervention or in combination with a non-digital/online component was included. The platform may include websites, mobile apps, SMS, and other online mediums. Interventions were excluded if they were telephone-based or included phone coaching or in-person face-to-face interventions without a digital/online component. The digital component of interest was the use of interactive multimedia content in the interventions for visual storytelling and/or play-based learning. Examples include the use of games, gamification, music, and/or animation. Given the novelty of this research area, studies were included where authors reported the use of a digital and/or online platform, and a standard data extraction template was used to collect information related to the use of interactive multimedia content included in the interventions.

#### 2.2.4. Outcomes

The main outcomes were any measure of the change in a child’s weight, BMI, waist circumference, diet, physical activity, sedentary behavior, screen time, and/or sleep from baseline to the last available follow-up, to assess intervention impact on child obesity-related anthropometric and/or behavioral health outcomes. Most of the outcome variables were continuous data, such as BMI and waist circumference, and were expressed as mean difference and standard deviation, and/or confidence intervals. Where categorical outcome data were reported, for example, whether participants improved dietary habits by eating breakfast every day, the effect measures were expressed as the proportion of participants who ate breakfast every day in the intervention group vs the control group.

#### 2.2.5. Settings

Studies undertaken in family-based or home-based settings were included. School-based and hospital-based studies and experiments in a controlled environment were excluded.

### 2.3. Study Selection

Two review authors (Leila Fathi and either Rebecca Farletti or Li Kheng Chai) screened abstracts and titles independently for eligible studies. Review authors were not blinded to author or journal information. The screening was performed using a standardized screening tool developed by the author team experienced in conducting systematic reviews based on the Cochrane Handbook for Systematic Reviews of Interventions [12], with reference to the Rapid Reviews to Strengthen Health Policy and Systems: a Practical Guide published by World Health Organization (WHO) [13]. The full texts of potentially eligible studies were retrieved for further screening. Conflicts between review authors regarding study eligibility were resolved by a third review author.

### 2.4. Data Extraction

Data from the included trials were extracted and checked by two review authors (Leila Fathi and Rebecca Farletti). Conflicts between review authors regarding extracted data were resolved by consensus and via a third review author where required. Extracted data included study characteristics (aims and objectives, study design, number of experimental conditions, targeted participants demographic characteristics, country, intervention description and duration, behavior change theory/techniques used, and overall conclusion); study outcomes, including the data collection method, the validity of instruments/measures used, effect size and measures of outcome variability; and source(s) of research funding and potential conflicts of interest.

### 2.5. Quality Appraisal

Critical appraisal was performed by two review authors (Li Kheng Chai and Robyn Littlewood) independently using the Joanna Briggs Institute Critical Appraisal Tools, including Checklist for Randomized Controlled Trials [14]. The critical appraisal tool that has previously been used by the author team in other systematic reviews, was adapted for use in this review and was piloted before use. A risk of bias classification (“high”, “low” or “unclear”) was assigned for each of the following study characteristics: sequence generation, allocation concealment, blinding of participants and personnel, blinding of outcome assessors, incomplete outcome data, selective outcome reporting, outcome measure reliability, trial design, and “other” potential sources of bias. Additionally, a criterion for “potential confounding” was included for the assessment of the risk of bias in non-randomized trial designs. An overall risk of bias classification was assigned to each study, giving consideration to all such study characteristics. Conflicts between review authors regarding the methodological quality of studies were resolved by consensus and via a third review author where required.

### 2.6. Data Synthesis

It was anticipated that differences in measures and study outcomes reported in the included studies may preclude the use of summary statistics to describe treatment effects and also necessitate a narrative synthesis. However, whenever possible, the relevant outcomes were synthesized and the effect measures were summarized including the child’s weight, BMI, waist circumference, and other anthropometry outcomes; diet, physical activity, sedentary behavior, sleep, and other obesity-related behavioral outcomes.

## 3. Results

### 3.1. Study Selection

**Figure 1:**
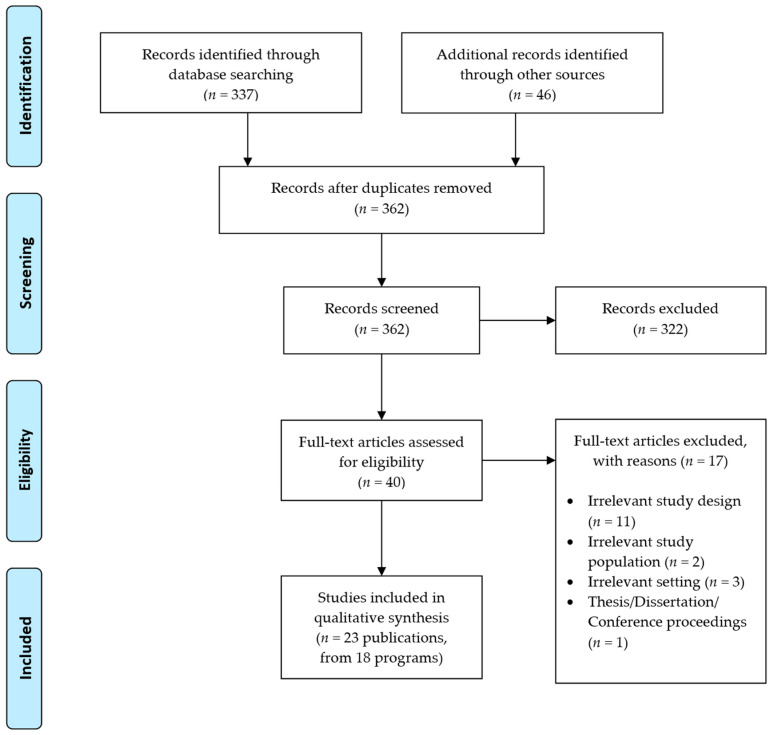
PRISMA 2009 Flow Diagram.

The study selection process is presented in an adapted Preferred Reporting Items for Systematic Reviews and Meta-Analyses (PRISMA) flow diagram [15] presented in Figure 1. The database searches identified 362 records for the title and abstract screening against the inclusion criteria, followed by 40 full texts retrieved for further screening, which resulted in the final inclusion of 23 publications (from 18 interventions) in this review. The majority of the 17 excluded publications were primary studies with irrelevant study designs (*n* = 11). A list of excluded studies and reasons for exclusion are presented in Supplementary Table S2 (see supplementary materials).

Web searching identified five additional digital interventions for obesity prevention and treatment in Australian children. Brief descriptions and characteristics of these interventions are presented in Supplementary Table S3 (see supplementary materials). The interventions predominantly used websites, telehealth coaching, and mobile apps as digital delivery methods. Only one of these interventions, i.e., the Kurbo app by WW International (formerly known as Weight Watchers International) has published evaluation findings; however, the study was a retrospective cohort study without a comparison arm [16]. The searches did not retrieve any other peer-reviewed publications which reported the acceptability, usability, or effectiveness of these interventions on obesity-related outcomes. As such, these interventions were excluded from the synthesis.

### 3.2. Quality Appraisal

Table 1 presents the results of the quality appraisal for the 18 interventions [17,18,19,20,21,22,23,24,25,26,27,28,29,30,31,32,33,34] included in the review. Overall, the majority of the studies were of moderate to high quality (i.e., meeting at least nine of the thirteen criteria). The studies have reported an adequate randomization procedure (*n* = 13) and concealment of group allocation (*n* = 11), while the two studies did not describe the randomization procedure, nor the allocation concealment. Blinding of participants was not reported in eight studies and deemed not possible in four studies due to the nature of the intervention study design (e.g., waitlist control). Only six studies were rated low in risk of bias related to blinding of participants to treatment assignment.

Blinding of intervention providers was not applicable to three studies where the interventions were web-based modules accessible by participants independently without needing an intervention provider. Additionally, blinding of intervention providers was not possible in six studies due to the nature of the study design (e.g., waitlist control commenced intervention at a later time). The blinding of outcome assessors was mixed in the included studies, where five studies were rated a high risk of bias and six studies were rated as unclear. In studies where BMI was reported, actual height and weight measurements were recorded by research staff using standardized protocols. However, one included study, conducted by Rangelov et al., used parent-reported child height and weight and presented outcomes as a percentage of weight categories (e.g., % healthy weight, % overweight/obesity) instead of BMI changes. All studies used a validated instrument tool when measuring weight, physical activity, diet, sedentary behavior, and/or sleep. The intention-to-treat statistical approach was applied in 11 studies using the last observation carried forward or multiple imputation method, including two studies that had 22% and 59% drop out rates, respectively.

### 3.3. Study Characteristics

All studies included parent-child dyads with the majority of children (*n* = 1693 of 2053) aged between 9–11 years. One study specifically focused on mothers [25], while another focused on fathers [27]. Among the included interventions, 10 were “treatment focused” that targeted children who were overweight or have obesity, and the remaining eight were “prevention focused” that targeted children in all weight categories, including one intervention that targeted children in the healthy weight category. Most of the studies (*n* = 13) were published in the last five years. The studies were conducted in eight countries including Belgium, Malaysia, Sweden, the USA, New Zealand, Australia, Canada, and Switzerland. Overall, the study period ranged between 4 and 20 weeks in duration, with the longest follow-up being between two months and two years from baseline. A total of 14 studies reported ≤ 20% dropout rate at follow-up, and the remaining four studies had a dropout rate that ranged between 22% to 59%. Demographic characteristics of families included in the studies and a summary of interventions are presented in Table 2. Further details on study aims, participant characteristics, attrition rates, intervention use, and theoretical framework are presented in Supplementary Table S4 (see supplementary materials).

### 3.4. Theoretical Framework

Behavior change theories (BCT) were used in 13 interventions with “Social Cognitive Theory” (SCT) applied most frequently (*n* = 9) [17,18,19,22,23,25,26,27,30]. The SCT theorizes behavioral outcomes (e.g., child behavior change) as a reciprocal interaction between the person (e.g., child) and environmental factors (e.g., home and family unit) [17]. Interventions focused on elements of behavior capability in parents, such as self-monitoring, goal setting, self-efficacy, problem-solving, relapse prevention, and stimulus control [17,18,23]. Similarly, a study by Knowlden et al. focused on the environment, emotional coping, expectations, self-control, and self-efficacy of parents [25]. Other interventions also focused on creating a shift in attitudes toward healthy behavior among parents [22] or the family unit [23]. Ahmad et al. encouraged parents to practice authoritative parenting skills, consistent with Wald et al., and focus on self-efficacy of the child’s healthy behaviors in addition to the elements of behavior capability in parents [17,33].

Nine studies incorporated one or more other behavior change theories, some in conjunction with SCT, when designing the intervention [22,23,26,27,30]. For example, Jake et al. incorporated elements of Family Systems Theory with SCT and conceptualized parent–child relationships in the context of reciprocal interactions [23]. Morgan et al. and Wald et al. incorporated Self-Determination Theory, which suggests that three basic psychological needs (autonomy, competency, and relatedness) must be satisfied to foster well-being [27,33]. Chai et al. and Perdew et al. used a different behavior change framework underpinned by constructs similar to SCT: attitude, motivation, self-efficacy, opportunity, behavioral regulation, identity, and habit [20]. Rangelov et al. used the Social Marketing Framework incorporating all six elements of the marketing mix: product, place, price, promotion, policy, and partnership.

The use of BCT was not reported in five studies [21,24,31,32,34]; however, two of the studies [32,34] described intervention strategies that were aligned with SCT, including goal setting, reinforcement, modeling, changing the home environment, problem-solving, and/or behavioral contracting.

### 3.5. Intervention Components and Usage

Interventions included in this rapid review facilitated delivery via websites (*n* = 11) [18,20,21,23,24,26,28,29,30,33,34], text messaging (*n* = 5) [18,20,24,29,31], Facebook (*n* = 3) [17,18,20], mobile apps (*n* = 2) [24,31] and/or video gaming (*n* = 2) [30,32]. Websites were used for providing additional resources to complement the in-person group sessions on topics, such as healthy living, physical activity, and recipe ideas [28,34]. Some intervention websites included an online discussion forum [25,28,29,33] to engage parents in sharing their challenges and successes while offering a forum for parents to ask questions [33]. Websites were also used for goal setting and self-monitoring, where families may log activities and track progress with an interactive graph [23,34]. Jake et al. reported high adherence to self-monitoring protocols with parents and children using the website for step and food logs [23]. A study by Williamson et al. provided instant feedback after parents completed online worksheets and quizzes involving self-reported health behavior [34].

Intervention websites provided content in the form of short videos [22], audiovisual presentations [25], or narrated graphic stories [21]. Studies reported moderate to high utilization of websites, ranging from around 86% to 93% of participants accessing the content [21,22,23], except for Rangelov et al. who reported 39% to 46% of participants visiting the website despite weekly email and SMS reminders. Evidence suggests that websites with interactive features have higher engagement rates compared to those with non-interactive content (e.g., texts and pictures) [34], which may explain the lower website usage rates in Rangelov et al.

An emerging number of studies have used apps and/or social media platforms to boost participant engagement with the intervention content. Two interventions used a combination of website, social media platforms, and text messaging which achieved high utilization rates with 77% to 86% of families accessing the website regularly [18,20]. Tuesdays had the highest number of website visits and 16:00 was the most popular time on most days [20]. Many parents also preferred a weekly text message on earlier days of the week, such as Monday (40%) and Tuesday (36%) [18]. To encourage long-term behavior maintenance, a study provided families with access to an app at the conclusion of the in-person group program, which included a variety of fun physical activities for families to complete and track together weekly. The app was used by 83% of families [27]. Another study [31] reported that 71% of families used the app at least two times per week, including 35% who used it at least three times a week. Studies reported higher rates of participation in WhatsApp and/or Facebook sessions compared to in-person sessions and websites [18]; indicating that a social media approach may enhance participation. Some parents also noted interaction among Facebook participants as an important feature [18].

Other interventions have used technological devices including a screentime monitoring device to help budget family media use (but 46% of parents reported never using the device to budget their child’s television or computer use) [26], and a Bluetooth weighing scale with no displays to indicate weight readings together, with a wrist-worn activity monitor that was connected to a gamified app that encourages physical activity (in which 80% of children participated) [24]. Two other interventions incorporated gamification in the form of video games and reported 76% to 91% participation rates [32].

### 3.6. Intervention Effects

A summary of intervention effects on child outcomes, including anthropometrics, dietary intake, physical activity level, sedentary behavior, screen time, and sleep, are presented in Table S5 (see supplementary materials). Overall, studies reported changes in BMI (*n* = 11 studies) [17,18,19,20,24,26,27,28,32,33,34], dietary intake (*n* = 11) [18,19,20,21,22,23,25,28,29,30,34], physical activity (*n* = 10) [19,20,22,23,25,26,27,28,31,32] and/or screen time (*n* = 6) in children [22,25,26,27,28,33]. Significant improvements were reported for the dietary intake (*n* = 5) [18,20,25,29,30] or physical activity levels (*n* = 4) of children [25,27,28,32]. Two [25,27] of six interventions were effective in reducing screen time. Overall, the attrition rates of included studies ranged between 9% and 59%, at the longest follow-up.

#### 3.6.1. Anthropometry

Studies predominantly reported anthropometric outcomes in BMI, BMI z-scores (zBMI), waist circumference, and body fat percentage. Intervention effects on child anthropometry reported in the included studies were mixed. Eight studies (*n* = 587 families) [18,19,20,26,27,28,33,34] reported that the changes in BMI, zBMI, and/or waist circumference were not significant. Only three studies (*n* = 207) [17,24,32] reported that BMI z-scores were significantly reduced in the intervention group compared to the control group and all these three studies were ‘treatment focused’ with one of these studies (*n* = 122) [17] also reported significant decreases in waist circumference percentile and body fat percentage in the intervention group. 

#### 3.6.2. Dietary Intakes

Overall, 11 studies have reported dietary outcome measures via food group consumption and total energy intake. Fruit and/or vegetable intakes were the most frequently reported in the studies [18,19,20,21,22,23,25,28,29,30], followed by sugar-sweetened beverages (SSB) [19,21,22,23,25,28,29]. Studies reported food and/or drink intakes in varying units, including frequency per day, frequency per week, portions per week, or standard serves per week. Mixed findings were reported on child dietary behavior where six studies (*n* = 313) [19,21,22,23,28,34] reported that the changes in fruit, vegetable, and/or SSB intakes were not significant compared to five studies that reported significant improvements. Intervention groups in four studies (*n* = 533) [18,20,25,30] showed a significant increase in healthy foods/food group consumption post-intervention with a decrease in SSB or discretionary food (e.g., fast food) intake. One study (*n* = 608) [29] found that families who received an SMS intervention had reported improved dietary intake for vegetables, but not for fruit, sweets, SSB, and water.

#### 3.6.3. Physical Activity

Among the 10 studies which measured physical activity, the six studies (*n* = 433) [19,20,22,23,26,31] reporting intervention effects were not significant when comparing the physical activity scores, step counts, or minutes of active transportation between groups. Four studies (*n* = 297) [25,27,28,32] reported a significant increase in physical activity levels or step counts post-intervention.

#### 3.6.4. Screen Time/Sedentary Behavior

Two of the six studies [22,25,26,27,28,33] that reported child screen time showed a significant intervention effect [25,27] on decreasing screen time at post-intervention and follow-up. The two studies (*n* = 175) [25,27] were delivered as a knowledge-based healthy lifestyle intervention focusing on one or more changed behaviors and they each included a weekly group session and web-based materials. While two other studies (*n* = 92) [28,33] used similar intervention modalities (i.e., weekly group sessions and websites), the changes in screen time were not significant.

#### 3.6.5. Sleep

Sleep outcome was reported in only one study [26] that focused on reducing leisure time screen-based sedentary behavior in children (*n* = 238) aged nine to 12 years, delivered over 20 weeks. A key focus was to train the primary caregivers to initiate change in the home environment, implementing behavior change strategies with an aim of facilitating behavioral change in the child. However, the changes in sleep duration (minutes/day) between groups from baseline to six months follow-up were not significant. This lack of effect may be explained by the bias associated with self-report measures and/or poor compliance, where 46% of parents reported never using the screen time monitoring device to budget their child’s television or computer use [26].

## 4. Discussion

The COVID-19 pandemic has impelled the potential of using online platforms for day-to-day tasks, such as remote work, videoconferencing, telehealth, and food ordering services. Online platforms and digital solutions are no longer unfamiliar to many and have increasingly demonstrated the potential for use in health promotion and prevention. This rapid review aimed to summarize the impact of family-based digital interventions for obesity prevention and treatment, with a focus on diet, physical activity, sedentary behavior, sleep, and/or weight-related outcomes in primary school-aged children.

Digital intervention for family-based childhood obesity prevention and treatment is an emerging research area with increased publications over the last five years. The interventions have shown promising preliminary results with modest improvement in child BMI, diet, and physical activity. Similar findings were reported in a systematic review [35] that examined the effectiveness of mobile apps aimed at obesity prevention in children aged eight to 12 years and suggested that apps for health behavior promotion interventions may potentially improve health behaviors among children, but their effectiveness in improving anthropometric measures remains unclear. When comparing the results between studies, it is important to consider the various quantifying units used in measuring dietary intake and physical activity. The lack of consistency in measuring and reporting approaches in behavioral studies such as these has hindered further analysis to be conducted to make a comprehensive review.

A scoping review [36] of E&M Health interventions (defined as the use of information and communications technology, especially Internet (web), text messages with SMS, smartphone apps, and social media to monitor, improve or enable health behavior or health care) for childhood obesity reported that weight, physical activity, and diet were frequently assessed, while limited reviews have focused on sedentary behavior. The current rapid review included sedentary behavior and found six studies that reported child screen time, in which two of the studies showed significant screen time reduction after the intervention. However, the evidence around screen time and sleep remains scarce. Only one study [26] included in the current review has reported sleep outcomes but the between-group differences were not significant. Similarly, a systematic review identified that only eight trials included a sleep component in obesity prevention interventions and that evidence on sleep intervention research is limited. Thus, the potential impact of sleep on childhood obesity remains unclear [11].

Interventions included in this review used websites, text messaging, Facebook, mobile apps, and/or video games, incorporating digital interactive elements and/or gamification. Research suggests that virtual delivery utilizing gamification can be effective in increasing the acceptability and accessibility of health interventions, thus, more readily supporting children and families to adopt healthy lifestyle changes [5]. A systematic review found that gamification, behavioral monitoring, and goal setting were common features of mobile apps aimed at childhood obesity prevention [35]. However, the reporting of process indicators, such as attendance tracking, participation, and retention rates were limited [35]. This rapid review found digital approaches are acceptable and positively received by families, with high participation rates.

There was emerging evidence of the use of social media and gamification to support program delivery. In line with established theories of intrinsic motivation [37], interactive digital components can employ motivational features such as goal setting, and real-time feedback through interface elements such as point scores, badges, levels, challenges, social support through leaderboards, teams and communication functions, and autonomy through customizable profiles and user choice in goals and activities [38,39]. Another study found that interventions using gamification to target nutrition behaviors among children and adolescents increased program adherence, knowledge, self-efficacy, and nutrition-related health behavior in the short term [39]. Similarly, interactive social media interventions reported improvements in overall wellbeing and were effective in improving physical activity among adults [40]. However, the effectiveness, dose of meaningful engagement (e.g., click-through rates, posts, comments), and sustainability of these novel approaches require further studies, given the limited existing evidence [41].

Despite several digital interventions for childhood obesity that were accessible in online marketplaces to the wider public in Australia, only one program targeted prevention while the remaining four programs focused on weight loss treatment or weight management. It is unclear whether the market programs have peer-reviewed evidence or rigorous evaluation to support their usability, acceptability, or effectiveness. This raises concerns regarding the quality and credibility of readily accessible programs on the internet. The limited evaluation findings of existing programs present a missed opportunity to advance the evidence base around digital interventions for childhood obesity, exchange learnings between peers, and inform future interventions. Thus, there is a research-practice gap that limits the availability of evidence-based childhood obesity prevention and treatment programs in the public domain, and more support is needed to enhance the translation of effective interventions into population health benefits.

A limitation of the current rapid review was the inclusion of just four electronic databases in the search strategy, unlike systematic reviews which usually search more databases. The search terms around digital and interactive interventions used in this rapid review were broad and less exhaustive, however, the lack of standard definition in this emerging field has contributed to the complication. Despite the rapid review design, the methodological rigor was upheld by including the quality appraisal of the included studies and the use of two independent reviewers across the screening phases, while applying the WHO practical guide for rapid reviews [13]. A strength of the current rapid review was the web searching of marketplace digital interventions for obesity prevention and treatment in children which may not have been indexed or published in the databases searched. The current rapid review also included sedentary behavior and sleep which were rarely included or reported in reviews. The rapid review also reported on the digital components of interventions and process indicators, such as intervention usage and engagement rates where available, instead of focusing solely on the efficacy results regarding behavior change which were commonly reported in other reviews.

Australia has committed to the WHO global target to halt the rise in obesity and being overweight, and recently released the National Obesity Strategy—a 10-year framework for action to prevent, reduce, and treat obesity and being overweight in Australia. The strategy has been agreed upon by Australian State and Territory Governments and has advocated for early intervention services to consider various delivery modes, including telehealth and other digital technology, that are affordable and accessible for all. For example, the Queensland Government has established a dedicated prevention agency, Health and Wellbeing Queensland, which will lead the statewide implementation of the National Obesity Strategy and drive innovative solutions to combat obesity. Evidence presented in the current rapid review supported the recommendations for virtual intervention delivery utilizing digital approaches, interactive elements, and/or gamification. However, more research is warranted to better understand how virtual delivery may impact screen time and sedentary behavior, the optimal dose and engagement for health behavior change, and whether educational screen time and discretionary screen time contribute to health and behavior outcomes differently. Future research assessing the effectiveness of digital interventions on improving sleep is needed, considering the limited research despite evidence suggesting sleep is associated with overweight and obesity in children [11].

## 5. Conclusions

In conclusion, family-based digital interventions for childhood obesity prevention and treatment were acceptable and well-received by families. The findings of the review suggest that digital interventions have shown some improvements in child BMI, diet, and physical activity, however, statistically significant results were mixed. The evidence around screen time and sleep remains scarce due to limited studies available. There is emerging evidence of the use of social media and gamification in digital interventions. However, further research is warranted to understand its long-term effectiveness and sustainability, and whether a blend of virtual and in-person approaches could be the recommended best practice for the future. Such approaches must consider the need for prevention and treatment interventions to be adaptable in response to unforeseen crises, such as a global pandemic.

## Figures and Tables

**Table 1:** Quality appraisal.

Author (Year)	Q1	Q2	Q3	Q4	Q5	Q6	Q7	Q8	Q9	Q10	Q11	Q12	Q13
Ahmad (2018) [17]	Y	Y	Y	Y	NA	Y	Y	Y	Y	Y	Y	Y	Y
Bakirci-Taylor (2019) [18]	Y	Y	Y	N	Y	U	Y	Y	N	Y	Y	Y	Y
Baranowski (2003) [19]	Y	Y	N	U	NA	U	N	U	U	Y	Y	Y	Y
Chai (2021) [20]	Y	Y	Y	U	Y	Y	Y	Y	Y	Y	Y	Y	Y
Cullen (2017) [21]	Y	U	Y	U	NA	U	Y	Y	N	Y	Y	Y	Y
De Lepeleere (2017) [22]	N	N	Y	NA	NA	Y	Y	Y	N	Y	Y	Y	N
Jake-Schoffman (2018) [23]	U	Y	U	U	U	Y	Y	N	U	Y	Y	Y	Y
Johansson (2020) [24]	Y	Y	Y	N	N	N	Y	Y	Y	Y	Y	Y	Y
Knowlden (2015) [25]	Y	U	Y	Y	U	Y	Y	Y	N	Y	Y	Y	Y
Maddison (2014) [26]	Y	Y	Y	NA	NA	U	Y	U	Y	Y	Y	Y	Y
Morgan (2019) [27]	Y	Y	U	NA	NA	N	Y	Y	Y	Y	Y	Y	Y
Perdew (2021) [28]	N	N	Y	NA	NA	N	Y	U	Y	Y	Y	Y	N
Rangelov (2018) [29]	Y	U	Y	U	Y	U	Y	Y	U	Y	Y	Y	Y
Thompson (2015) [30]	Y	Y	Y	Y	Y	Y	Y	Y	U	Y	Y	Y	Y
Trost (2021) [31]	Y	Y	Y	U	Y	Y	Y	Y	Y	Y	Y	Y	Y
Trost (2014) [32]	Y	U	Y	U	N	N	Y	Y	Y	Y	Y	Y	Y
Wald (2018) [33]	U	Y	Y	U	NA	U	U	Y	Y	Y	U	Y	Y
Williamson (2005) [34]	U	U	Y	Y	NA	N	Y	Y	Y	Y	Y	Y	Y

Y: Yes, N: No, U: Unclear, NA: Not Applicable. Q1. Was true randomization used for the assignment of participants to treatment groups? Q2. Was allocation to treatment groups concealed? Q3. Were treatment groups similar at the baseline? Q4. Were participants blind to the treatment assignment? Q5. Were those delivering treatment blind to treatment assignment? Q6. Were outcome assessors blind to the treatment assignment? Q7. Were treatment groups treated identically other than the intervention of interest? Q8. Was the follow-up complete and if not, were differences between groups in terms of their follow-up adequately described and analyzed? Q9. Were participants analyzed in the groups to which they were randomized? Q10. Were outcomes measured in the same way for treatment groups? Q11. Were outcomes measured in a reliable way? Q12. Was appropriate statistical analysis used? Q13. Was the trial design appropriate, and was any deviation from the standard RCT design (individual randomization, parallel groups) accounted for in the conduct and analysis of the trial?

**Table 2:** Summary of intervention studies and key findings.

Author (Year) Country	Prevention/Treatment ^a^	Study Design	Participant Characteristics	Intervention Duration and Intensity	Digital Components	Overall Findings ^b^
Ahmad (2018) Malaysia [17]	Treatment	RCT	134 parent-child dyads. Children aged 7–10 years. All participants were Malay females.	4 weeks of weekly training for parents to change child behavior and 3 months of weekly booster which consisted of weekly one-hour sessions using a WhatsApp group.	▪ Face-to-face session (week 1 and 4), subsequently uploaded to Facebook ▪ Sessions on Facebook Group (week 2 and 3) ▪ WhatsApp group for information sharing	BMI z-scores were significantly reduced in the intervention group compared to the wait-list group for all the children at 6-month post-training. For waist circumference percentile and body fat percentage, the intervention group experienced a significant reduction compared to the wait-list group, within the obese subgroup, and within the overweight subgroup.
Bakirci-Taylor (2019) USA [18]	Prevention	RCT	30 parent-child dyads. Children were aged 3–8 years. All parents were female, married, and predominantly Caucasian. Over 70% had at least a bachelor’s degree and 40% reported incomes of ≥$75,000.	10 weeks of the mobile Jump2Health intervention which included 3 components: a mobile website (Jump2Health), social media (Facebook page), and short message service or text messages. The Facebook page provided information that was unavailable on the mobile Jump2Health website, but it also Mentioned and reinforced information and text found on the website and promoted linked resources on the website.	▪ Website (main content) ▪ Facebook page (additional content) ▪ Mobile text messages (about FV consumption)	Skin carotenoids of both children and parents showed significant Week x Treatment interactions in the INT group compared with CON (*p* < 0.001) indicating increased veg intake.
Baranowski (2003) USA [19]	Prevention	RCT	35 parent-child dyads. Children aged 8 years with a BMI in the 50th percentile for age and gender specific BMI. All participants were female African Americans. Majority had a household income of > $40,000, college graduate, or higher education.	4-week summer day camp, followed by an 8-week home Internet intervention for the girls and their parents which included weekly behavioral/environmental foci. The treatment camp blended usual camp activities with activities specially designed for *GEMS-FFFP.*	▪ Summer day camp (4 weeks in duration) ▪ Website (new content weekly for 8 weeks) ▪ Weekly email and phone reminders to log on	There were no significant changes in BMI, waist circumference, dietary intake, and physical activity level.
Chai (2021) Australia [20]	Treatment	RCT	46 parent-child dyads. Children (mean age 9 years) were predominantly male, overweight/obese and resided with both biological parents. Most parents (mean age 41 years) were female, of middle SES, living in major cities, overweight/obese, and attained certificate/diploma or postgraduate degree.	Both INT 1 and 2 groups received two telehealth consultations delivered by a dietitian, 12 weeks access to a nutrition website and a private Facebook group. INT 2 group received additional text messages.	▪ Website (new content weekly for 12 weeks). ▪ Telehealth dietitian (2 appointments) ▪ Facebook group ▪ Mobile text messages (various frequency for 12 weeks	Percentage energy from EDNP food was reduced and percentage energy from nutrient-rich core food was increased in *Telehealth+SMS* when compared to CON.
Cullen (2017) USA [21]	Prevention	RCT	126 parent-child dyads. Children aged 8–12 years. All participants were African American. Majority of parents were female, aged < 40 years, college graduate or higher education, and have less than two children.	Approximately 8 weeks web-based program for African American families that was designed to promote healthy home food environments, positive parental behaviors related to improving dietary behaviors of family members, and goal setting.	▪ Website (narrated graphic story viewing). ▪ Eight stories follow the Johnson family (an African American family with two 8- to 12-year-old children) as they try to develop healthier eating habits. ▪ After viewing the weekly story, the parents had a challenge (goal) to complete during the next week and viewed a family food problem. Tip sheets targeting the session content and recipes could be downloaded from the website.	Home availability of juice (*p* < 0.05), vegetables (*p* < 0.01), and low-fat/fat-free foods (*p* < 0.05) were significantly higher in INT at 2 months. Parent menu planning skills were significantly higher in INT at 6 months. Both INT and CON groups showed significant increases in home juice/fruit availability, parent modelling, food preparation practices, and menu planning, and a significant decrease in home sugar-sweetened beverage availability (all *p* < 0.05).
De Lepeleere (2017) Belgium [22]	Prevention	Quasi-experimental controlled trial	135 parents of a primary school-aged child. Majority of parents were female and from a medium-high SES.	4-week access to website (health promoting videos); content delivered weekly over four weeks; contact time ~2 min per video (22 videos)	▪ Website (online videos followed by narrator explanation) ▪ 22 short videos (2 min each) showing a difficult child-parent scenario followed by an appropriate reaction of the parent, then a narrator explains the parenting practices showed in the video.	Most significant intervention effects were found for more complex parenting practices (e.g., an increase in motivating the child to eat fruit). Subgroup analyses showed that the intervention had more effect on the actual parenting practices related to PA, screen-time, and healthy diet in parents of older children (10–12 years old), whereas intervention effects on parental self-efficacy related to those behaviors were stronger in parents of younger children (6–9 years).
Jake-Schoffman (2018) USA [23]	Prevention	RCT	33 parent-child dyads. Majority of parents were female, Caucasian, college graduates, and have obesity. Majority of children were female, Caucasian, aged 11 years, and have a healthy weight.	Dyads were asked to self-monitor using a mobile responsive design website made for the intervention for 12 weeks.	▪ Mobile website with messaging function and features such as side-by-side graphs to show the daily progress of parents and children toward study goals, and a messaging feature where parents and children could send messages of support and encouragement to one another to help reinforce behavioral goals. ▪ Websites included sections directed to parents, separate sections for children, and a section for the family, to encourage collaboration. ▪ Email newsletter	There were no significant Group × Time × Parent or Group × Time effects on any of the intervention outcomes: minutes of MVPA (accelerometer), daily steps (pedometer), servings of fruit, vegetables, fast food, and SSBs.
Johansson (2020) Sweden [24]	Treatment	RCT	28 children aged 5–12 years who have obesity according to the International Obesity Task Force (IOTF) with parents who speak Swedish.	6 months daily self-monitoring of weight recorded via a mobile app used by parents, a website in which clinicians could track treatment progress, prespecified treatment goals for change in degree of obesity shown in the app and on the website, and text message interactions between clinicians and parents. In addition to the mHealth approach, the intervention group received standard care (clinical appointment).	▪ Daily weighing at home on scales with no displays to indicate weight and data were transferred to the mobile app (for families) and to the clinic’s interface (website). ▪ The clinicians were instructed to check the participants’ weight charts on the clinic’s interface at least weekly and give feedback via text messages. ▪ A wrist-worn activity monitor was connected to a gamified app which prompted for physical activity to generate rewards that were displayed in the app.	At 6 months the intervention group had a greater reduction in standardized BMI than standard care.
Knowlden (2015) USA [25]	Prevention	RCT	57 mothers with children aged 4–6 years. Mothers (mean age of 36 years) were predominantly Caucasian, married, unemployed/homemakers. Children (mean age of 5 years) were primarily Caucasian, male, with a mean age of 5 years.	4 weekly audiovisual presentations (30 min per session) via website and 1 booster session.	▪ Each of the five online sessions included a 10- to-15-min audiovisual presentation, an interactive online worksheet, and a discussion board post designed to increase knowledge supplemented each module.	The EMPOWER arm of the trial resulted in an overall increase of 1.680 daily cups of fruits and vegetables consumed by children, relative to the comparison group (*p* < 0.001, 95% confidence interval. Web-based maternal-facilitated interventions can induce sustained effects on child behaviors.
Maddison (2014) New Zealand [26]	Treatment	RCT	251 parent-child dyads. Children (mean age of 11 years) were predominantly male and of Pacific origin	Delivered over 20 weeks, consisting of a face-to-face meeting with the parent/caregiver and the child to deliver intervention content; TV monitoring device; monthly newsletters.	▪ Initial face-to-face session ▪ Website (health information and links to community-based activity programs) ▪ Time Machine TV monitoring device (2 devices per family) ▪ Activity pack for children ▪ The Time Machine was connected to a TV, or other media device (e.g., DVD player, video game console), but it was not possible to connect the device to a computer. Each Time Machine came with 30 tokens, with each token allowing 30 min of viewing time; however, caregivers were able to allocate these as they chose.	There was no significant difference in change of BMI z-scores between the intervention and control groups, although a favorable trend was observed (−0.016; 95% CI: −0.084, 0.051; *p* = 0.64). There were also no significant differences in secondary outcomes, except for a trend towards increased children’s moderate intensity physical activity in the intervention group (24.3 min/d; 95% CI: −0.94, 49.51; *p* = 0.06).
Morgan (2019) Australia [27]	Prevention	RCT	153 father-child dyads. Children were aged 4–12 years. Most fathers were employed, born in Australia, and were married or living with a partner (99%). Families were represented from most socio-economic areas.	90-min group sessions weekly for 8 weeks that included educational and practical components. They were provided with a web-based app at the conclusion of the program for long-term maintenance.	▪ 8 weekly group sessions (90 min) ▪ Printed resources ▪ App: To encourage long-term behavior maintenance, families were provided with access to a web-based app at the conclusion of the program, which included a variety of fun physical activities for daughters and fathers to complete and track together weekly.	ITT analyses revealed favorable group-by-time effects for physical activity in daughters (*p* = 0.02, d = 0.4) and fathers (*p* < 0.001, d = 0.7) at 9 months. At postintervention and follow-up, significant effects (*p* < 0.05) were also identified for daughters’ fundamental movement skills competence (objective: d = 1.1–1.2; perceived: d = 0.4–0.6), a range of fathers’ physical activity parenting practices (d = 0.3–0.8), and screen-time for daughters (d = 0.5–0.8) and fathers (d = 0.4–0.6, postintervention only). Program satisfaction and attendance were very high.
Perdew (2021) Canada [28]	Treatment	Quasi-experimental design	71 parent-child dyads. Children were aged 8–12 years; at or above 85th percentile for BMI for age and sex.	10 weekly face-to-face 90 min sessions, four community-based activities (i.e., family grocery store tour), and an interactive web-portal.	▪ 10 weekly face-to-face 90-min sessions, ▪ Four community-based activities (i.e., family grocery store tour), and ▪ Following the in-person sessions, 10 weekly online interactive lessons were made available to the families using a web portal.	Children’s BMI z-scores were not significantly changed. The intervention group significantly improved their days of moderate-to-vigorous physical activity relative to control; however, child dietary behaviors were not significantly changed. Relative to control, intervention group showed significant improvements in physical activity.
Rangelov (2018) Switzerland [29]	Prevention	RCT	608 parent-child dyads. Children (mean age of 8.5 years) were in the first two years of secondary school and about equal proportion of boys and girls.	8 weeks access to website (parents) and a personalized and tailored letter by post (children). The emails (INT 1) and mobile text messages (INT 2) were used as weekly reminders to prompt parents to visit the Website. The email also provided a short summary of the weekly theme.	▪ Website (weekly nutrition content and a forum for participants discussion together and with a dietitian) ▪ Emails and mobile text messages were sent as weekly reminders to prompt parents to visit the website.	Overall, the intervention effects were not significantly different across groups. Children increased their daily consumption of fruit and decreased that of sweets regardless of the group they were assigned.
Thompson (2015) USA [30]	Prevention	RCT	400 parent-child dyads. Children were in 4th or 5th grade (around 9–11 years). Almost evenly distributed by gender (female, 52.7%) and were of diverse ethnicity (White-36.8%, Hispanic 27.4%, African American 26.4%). Parents were mostly female (96.3%), White (40.3%), married (77.5%), and 40–59 years old (55.3%). Highest level of household education was predominately post-graduate study (36.7%), and average household income was >$61,000 (57.6%).	All INT groups played the 10-episode (1 h each) online videogame. The groups varied only in type of implementation intention created after setting a goal to eat FV, including the use of an action plan (specifying actions), or a coping plan (identifying barriers), or both action and coping plans.	▪ 10 episodes of online video game ▪ 10 newsletters (for parents) ▪ 10 installment (parents only) website (the parent intervention was connected to the child intervention.)	A significant group-by-time interaction for FV intake (*p* < 0.001) was found in only the *Action* group, which had significant increases in FV intake at post 1 (*p* < 0.0001) and post 2 (*p* < 0.0001). No other significant interactions were observed.
Trost (2021) Australia [31]	Prevention	RCT	34 parent-child dyads. Children aged 3–6 years and predominantly were Caucasian. Parents were college graduate or higher education.	Families were free to use the app ad libitum but were asked to complete a minimum of three Moovosity™ activities per week, over 8 weeks. Parents were sent mobile text messages and emails every two weeks.	▪ 8-week access to Moovosity™ mobile application (app) designed to promote the development of FMS and increase physical activity in young children ▪ Families were free to use the app ad libitum but were asked to complete a minimum of three Moovosity™ (The Kinetica Group Pty Ltd, South Melbourne, VIC, Australia) activities per week. ▪ Mobile text messages and Emails fortnightly reminders for use of the app	There were no significant intervention effects observed for child PA.
Trost (2014) USA [32]	Treatment	RCT	75 parent-child dyads. Children aged 8–12 years, had a BMI greater than the 85th percentile for sex and age and predominantly were female and White. Parents were college graduates or postgraduates.	INT group received hardware consisting of a game console and motion capture device and 1 active game at their second treatment session and a second game in week 9 of the program.	▪ Video games using a game console and motion capture device with 2 active sports games (at second treatment and week 9). ▪ INT program is 16 weekly group sessions.	Participants in the program and active gaming group exhibited significant increases in MVPA at week 16 (*p* < 0.05). In the program-only group, a decline or no change was observed in the moderate-to-vigorous and vigorous physical activity. Participants in both groups exhibited significant reductions in percentage overweight and BMI z scores at week 16. However, the program and active gaming group exhibited significantly greater reductions in percentage overweight.
Wald (2018) USA [33]	Treatment	RCT	73 parent-child dyads. Children (mean age 5 years) were predominantly non-Hispanic and overweight/obese. Mothers were predominantly aged < 40 years, college graduate or higher education, and married.	The INT was composed of 6-in-person group sessions and a customized website over 12 months.	▪ 6 weekly group counseling sessions ▪ 1-year access to Website with weekly update including health topics related to nutrition and physical activity, local resources for current activities for children and families, personal stories that emphasized authoritative parenting, interactive discussion group, and Ask the Expert. ▪ Parents were encouraged to share their triumphs and challenges so that all might benefit.	Among children with 12-month visits, BMI z-scores decreased from baseline to 12 months in both the control and intervention arms; however, the mean reductions were not significantly different between the control and intervention groups (*p* = 0.7492). The percent of children who reduced their screen time by ≥15% did not differ significantly between the intervention and control groups.
Williamson (2005) USA [34]	Treatment	RCT	57 African American girls aged 11–15 years.	Interactive website and 4 face-to-face sessions of behavior modification over 12 weeks focused on goal setting, behavioral contracting, monitoring of progress, and problem-solving. Participant-initiated weekly emails with counsellor.	▪ 4 face-to-face counselling sessions during the first 12 weeks of program. The intervention used a family-oriented format, i.e., a program that invited the parents, the child, and other members of the family to be involved using mutual problem-solving and behavioral contracting. ▪ Interactive website components included interactive graph to track exercise, interactive food monitoring worksheets with instant feedback on food choices, problem-solving worksheet (online), and a quiz followed every weekly lesson with instant feedback provided. ▪ Online counseling included weekly email communication with counsellor for feedback on program components (e.g., quizzes, lessons, weight graphs, goal setting, clinic appointments).	Participants in the intervention group lost significantly (*p* < 0.05) more body fat (−1.12 ± 0.47 SE) than the control group 0.43 ± 0.47 SE). There was a significant difference in BMI change between groups (intervention −0.19 ± 0.24 SE, < 0.05, control +0.65 ± 0.23 SE, *p* < 0.05). Participants in the intervention group significantly reduced fat intake compared with control group (FFQ) (−145.67 ± 37.67 SE, *p* < 0.05)

RCT: Randomized Controlled Trial; INT: intervention group; CON: control or comparison group; BMI: Body Mass Index; N/A: not applicable; NR: not reported; NS: not significant; FJV: fruit, juice, and vegetables; PA: physical activity; EDNP: energy dense nutrient poor; SSB: sugar-sweetened beverages; FV: fruit and vegetables; MVPA: moderate to vigorous physical activity; ITT: intention-to-treat. ^a^ Prevention studies had normal weight as participant inclusion criteria or no weight criteria specified. Treatment studies had overweight or obesity status criteria as participant inclusion criteria. ^b^ Between-group differences. Note: Refer to supplementary materials Supplementary Tables S4 and S5 for further details on study aims, participant characteristics, attrition rates, intervention use, theoretical framework, and outcomes related to anthropometry, dietary intake, physical activity, sedentary behaviour, screen time, and sleep.

## Data Availability

Not applicable.

## Supplementary Materials

The following supporting information can be downloaded at https://www.mdpi.com/article/10.3390/nu14224837/s1: Table S1: Search terms; Table S2: List of excluded studies and reasons for exclusion; Table S3: Interventions available on marketplace (identified from web searching); Table S4: Characteristics of included studies; Table S5: Intervention effects on child outcomes.
