# Supplementary material for: A Rapid Review of the Impact of Family-Based Digital Interventions for Obesity Prevention and Treatment on Obesity-Related Outcomes in Primary School-Aged Children
Source: Nutrients. 2022 Nov 15;14(22):4837. doi: 10.3390/nu14224837 (PMC9698336; doi:10.3390/nu14224837)
Supplement: Supplementary file 1 [file nutrients-14-04837-s001.zip › nutrients-2013900-supplementary.pdf]

nutrients

Supplementary Table S1. Search terms.

Database(s): Ovid MEDLINE(R) 1946 to 29 January 2021

Search Number	Searches
1	family.mp.
2	parent.mp.
3	carer.mp.
4	families.mp.
5	mother.mp.
6	father.mp.
7	1 or 2 or 3 or 4 or 5 or 6
8	child.mp.
9	children.mp.
10	school aged.mp.
11	young person.mp.
12	youth.mp.
13	kid*.mp.
14	paediatrics.mp.
15	pediatrics.mp.
16	infant.mp.
17	(8 or 9 or 10 or 11 or 12 or 13 or 14 or 15) not 16
18	online program.mp.
19	digital.mp.
20	multimedia.mp.
21	app.mp.
22	website.mp.
23	internet.mp.
24	web-based.mp.
25	web based.mp.
26	eHealth.mp.
27	mHealth.mp.
28	18 or 19 or 20 or 21 or 22 or 23 or 24 or 25 or 26 or 27
29	gaming.mp.
30	gamification.mp.
31	game.mp.
32	music.mp.

33	animat*.mp.
34	interactive.mp.
35	song.mp.
36	soundtrack.mp.
37	29 or 30 or 31 or 32 or 33 or 34 or 35 or 36
38	body mass index.mp.
39	body composition.mp.
40	waist circumference.mp.
41	nutrition.mp.
42	diet*.mp.
43	eating habit*.mp.
44	food consumption.mp.
45	food intake.mp.
46	physical activity.mp.
47	exercise.mp.
48	sedentary.mp.
49	sleep.mp.
50	obesity.mp.
51	weight.mp.
52	38 or 39 or 40 or 41 or 42 or 43 or 44 or 45 or 46 or 47 or 48 or 49 or 50 or 51
53	7 and 17 and 28 and 37 and 52

* Wildcard used in search terms to represent one or more other characters.

Database - CINAHL Complete

Last run via: Interface - EBSCOhost Research Databases

Limiters/Expanders: Limiters - Full Text; Expanders - Apply equivalent subjects; Search modes - Boolean/Phrase

Search Number	Query
S1	TI family OR AB family
S2	TI parent OR AB parent
S3	TI carer OR AB carer
S4	TI families OR AB families
S5	TI mother OR AB mother
S6	TI father OR AB father
S7	S1 OR S2 OR S3 OR S4 OR S5 OR S6
S8	TI child OR AB child
S9	TI children OR AB children
S10	TI 'school aged' OR AB 'school aged'
S11	TI 'young person' OR AB 'young person'
S12	TI youth OR AB youth

S13	TI kid* OR AB kid*
S14	TI paediatrics OR AB paediatrics
S15	TI pediatrics OR AB pediatrics
S16	TI infant OR AB infant
S17	(S8 OR S9 OR S10 OR S11 OR S12 OR S13 OR S14 OR S15) NOT S16
S18	TI 'online program' OR AB 'online program'
S19	TI digital OR AB digital
S20	TI multimedia OR AB multimedia
S21	TI app OR AB app
S22	TI website OR AB website
S23	TI internet OR AB internet
S24	TI web-based OR AB web-based
S25	TI 'web based' OR AB 'web based'
S26	TI eHealth OR AB eHealth
S27	TI mHealth OR AB mHealth
S28	S18 OR S19 OR S20 OR S21 OR S22 OR S23 OR S24 OR S25 OR S26 OR S27
S29	TI gaming OR AB gaming
S30	TI gamification OR AB gamification
S31	TI game OR AB game
S32	TI music OR AB music
S33	TI animat* OR AB animat*
S34	TI interactive OR AB interactive
S35	TI song OR AB song
S36	TI soundtrack OR AB soundtrack
S37	S29 OR S30 OR S31 OR S32 OR S33 OR S34 OR S35 OR S36
S38	TI 'body mass index' OR AB 'body mass index'
S39	TI 'body composition' OR AB 'body composition'
S40	TI 'waist circumference' OR AB 'waist circumference'
S41	TI nutrition OR AB nutrition
S42	TI diet* OR AB diet*
S43	TI 'eating habit*' OR AB 'eating habit*'
S44	TI 'food consumption' OR AB 'food consumption'
S45	TI 'food intake' OR AB 'food intake'
S46	TI 'physical activity' OR AB 'physical activity'
S47	TI exercise OR AB exercise
S48	TI sedentary OR AB sedentary
S49	TI sleep OR AB sleep
S50	TI obesity OR AB obesity
S51	TI weight OR AB weight
S52	S38 OR S39 OR S40 OR S41 OR S42 OR S43 OR S44 OR S45 OR S46 OR S47 OR S48 OR S49 OR S50 OR S51
S53	S7 AND S17 AND S28 AND S37 AND S52

* Wildcard used in search terms to represent one or more other characters.

Database - APA PsycInfo

Last run via: Interface - EBSCOhost Research Databases

Search Number	Searches
S1	TI family OR AB family
S2	TI parent OR AB parent
S3	TI carer OR AB carer
S4	TI families OR AB families
S5	TI mother OR AB mother
S6	TI father OR AB father
S7	S1 OR S2 OR S3 OR S4 OR S5 OR S6
S8	TI child OR AB child
S9	TI children OR AB children
S10	TI 'school aged' OR AB 'school aged'
S11	TI 'young person' OR AB 'young person'
S12	TI youth OR AB youth
S13	TI kid* OR AB kid*
S14	TI paediatrics OR AB paediatrics
S15	TI pediatrics OR AB pediatrics
S16	TI infant OR AB infant
S17	(S8 OR S9 OR S10 OR S11 OR S12 OR S13 OR S14 OR S15) NOT S16
S18	TI 'online program' OR AB 'online program'
S19	TI digital OR AB digital
S20	TI multimedia OR AB multimedia
S21	TI app OR AB app
S22	TI website OR AB website
S23	TI internet OR AB internet
S24	TI web-based OR AB web-based
S25	TI 'web based' OR AB 'web based'
S26	TI eHealth OR AB eHealth
S27	TI mHealth OR AB mHealth
S28	S18 OR S19 OR S20 OR S21 OR S22 OR S23 OR S24 OR S25 OR S26 OR S27
S29	TI gaming OR AB gaming
S30	TI gamification OR AB gamification
S31	TI game OR AB game
S32	TI music OR AB music
S33	TI animat* OR AB animat*
S34	TI interactive OR AB interactive
S35	TI song OR AB song
S36	TI soundtrack OR AB soundtrack
S37	S29 OR S30 OR S31 OR S32 OR S33 OR S34 OR S35 OR S36

S38	TI 'body mass index' OR AB 'body mass index'
S39	TI 'body composition' OR AB 'body composition'
S40	TI 'waist circumference' OR AB 'waist circumference'
S41	TI nutrition OR AB nutrition
S42	TI diet* OR AB diet*
S43	TI 'eating habit*' OR AB 'eating habit*'
S44	TI 'food consumption' OR AB 'food consumption'
S45	TI 'food intake' OR AB 'food intake'
S46	TI 'physical activity' OR AB 'physical activity'
S47	TI exercise OR AB exercise
S48	TI sedentary OR AB sedentary
S49	TI sleep OR AB sleep
S50	TI obesity OR AB obesity
S51	TI weight OR AB weight
S52	S38 OR S39 OR S40 OR S41 OR S42 OR S43 OR S44 OR S45 OR S46 OR S47 OR S48 OR S49 OR S50 OR S51
S53	S7 AND S17 AND S28 AND S37 AND S52

* Wildcard used in search terms to represent one or more other characters.

Cochrane Library

Search Number	Search
1	(family):ti,ab,kw (Word variations have been searched)
2	(parent):ti,ab,kw (Word variations have been searched)
3	(carer):ti,ab,kw (Word variations have been searched)
4	(families):ti,ab,kw (Word variations have been searched)
5	(mother):ti,ab,kw (Word variations have been searched)
6	(father):ti,ab,kw (Word variations have been searched)
7	MeSH descriptor: [Family] this term only
8	MeSH descriptor: [Parenting] explode all trees
9	MeSH descriptor: [Caregivers] explode all trees
10	MeSH descriptor: [Mothers] explode all trees
11	MeSH descriptor: [Fathers] explode all trees
12	1 or 2 or 3 or 4 or 5 or 6 or 7 or 8 or 9 or 10 or 11
13	(child):ti,ab,kw (Word variations have been searched)
14	(children):ti,ab,kw (Word variations have been searched)
15	("school aged"):ti,ab,kw (Word variations have been searched)
16	("young person"):ti,ab,kw (Word variations have been searched)
17	(youth):ti,ab,kw (Word variations have been searched)
18	(kid*):ti,ab,kw (Word variations have been searched)
19	(paediatrics):ti,ab,kw (Word variations have been searched)
20	(pediatrics):ti,ab,kw (Word variations have been searched)

21	MeSH descriptor: [Child] explode all trees
22	MeSH descriptor: [Adolescent] explode all trees
23	MeSH descriptor: [Pediatrics] explode all trees
24	(infant):ti,ab,kw (Word variations have been searched)
25	(13 or 14 or 15 or 16 or 17 or 18 or 19 or 20 or 21 or 22 or 23) not 24
26	("online program"):ti,ab,kw (Word variations have been searched)
27	(digital):ti,ab,kw (Word variations have been searched)
28	(multimedia):ti,ab,kw (Word variations have been searched)
29	(app):ti,ab,kw (Word variations have been searched)
30	(website):ti,ab,kw (Word variations have been searched)
31	(internet):ti,ab,kw (Word variations have been searched)
32	(web-based):ti,ab,kw (Word variations have been searched)
33	("web based"):ti,ab,kw (Word variations have been searched)
34	(eHealth):ti,ab,kw (Word variations have been searched)
35	(mHealth):ti,ab,kw (Word variations have been searched)
36	MeSH descriptor: [Internet-Based Intervention] explode all trees
37	MeSH descriptor: [Multimedia] explode all trees
38	MeSH descriptor: [Mobile Applications] explode all trees
39	MeSH descriptor: [Telemedicine] explode all trees
40	26 or 27 or 28 or 29 or 30 or 31 or 32 or 33 or 34 or 35 or 36 or 37 or 38 or 39
41	(gaming):ti,ab,kw (Word variations have been searched)
42	(gamification):ti,ab,kw (Word variations have been searched)
43	(game):ti,ab,kw (Word variations have been searched)
44	(music):ti,ab,kw (Word variations have been searched)
45	(animat*):ti,ab,kw (Word variations have been searched)
46	(interactive):ti,ab,kw (Word variations have been searched)
47	(song):ti,ab,kw (Word variations have been searched)
48	(soundtrack):ti,ab,kw (Word variations have been searched)
49	MeSH descriptor: [Video Games] explode all trees
50	MeSH descriptor: [Music] explode all trees
51	41 or 42 or 43 or 44 or 45 or 46 or 47 or 48 or 49 or 50
52	("body mass index"):ti,ab,kw (Word variations have been searched)
53	("body composition"):ti,ab,kw (Word variations have been searched)
54	("waist circumference"):ti,ab,kw (Word variations have been searched)
55	(nutrition):ti,ab,kw (Word variations have been searched)
56	(diet*):ti,ab,kw (Word variations have been searched)
57	("eating habit*"):ti,ab,kw (Word variations have been searched)
58	("food consumption"):ti,ab,kw (Word variations have been searched)
59	("food intake"):ti,ab,kw (Word variations have been searched)
60	("physical activity"):ti,ab,kw (Word variations have been searched)
61	(exercise):ti,ab,kw (Word variations have been searched)

62	(sedentary):ti,ab,kw (Word variations have been searched)
63	(sleep):ti,ab,kw (Word variations have been searched)
64	(obesity):ti,ab,kw (Word variations have been searched)
65	(weight):ti,ab,kw (Word variations have been searched)
66	MeSH descriptor: [Body Mass Index] explode all trees
67	MeSH descriptor: [Body Composition] explode all trees
68	MeSH descriptor: [Waist Circumference] explode all trees
69	MeSH descriptor: [Nutrition Assessment] explode all trees
70	MeSH descriptor: [Diet] explode all trees
71	MeSH descriptor: [Feeding Behaviour] explode all trees
72	MeSH descriptor: [Eating] explode all trees
73	MeSH descriptor: [Exercise] explode all trees
74	MeSH descriptor: [Sedentary Behaviour] explode all trees
75	MeSH descriptor: [Sleep] explode all trees
76	MeSH descriptor: [Obesity] explode all trees
77	MeSH descriptor: [Body Weight Changes] explode all trees
78	52 or 53 or 54 or 55 or 56 or 57 or 58 or 59 or 60 or 61 or 62 or 63 or 64 or 65 or 66 or 67 or 68 or 69 or 70 or 71 or 72 or 73 or 74 or 75 or 76 or 77
79	12 and 25 and 40 and 51 and 78

* Wildcard used in search terms to represent one or more other characters.

Supplementary Table S2. List of excluded studies and reasons for exclusion.

**Studies excluded**	Reasons for exclusion
Ahmad N. Effectiveness of Using Social Media in a Family-based Intervention for Childhood Obesity. [Trial registration number: ACTRN12617000844347] Available from: https://anzctr.org.au/Trial/Registration/TrialReview.aspx?id=372531&isReview=true (Accessed on 15 November 2022).	A
Aljafari A, Rice C, Gallagher JE, Hosey MT. An oral health education video game for high caries risk children: study protocol for a randomized controlled trial. Trials. 2015 Dec;16(1):1-0.	A
Bell BM, Martinez L, Gotsis M, Lane HC, Davis JN, Antunez-Castillo L, Ragusa G, Spruijt-Metz D. Virtual sprouts: a virtual gardening pilot intervention increases self-efficacy to cook and eat fruits and vegetables in minority youth. Games for health journal. 2018 Apr 1;7(2):127-35.	C
Calvert SL, Staiano AE, Bond BJ. Electronic gaming and the obesity crisis. New directions for child and adolescent development. 2013 Mar;2013(139):51-7.	A
Cunningham-Sabo L, Lohse B, Smith S, Browning R, Strutz E, Nigg C, Balgopal M, Kelly K, Ruder E. Fuel for Fun: a cluster-randomized controlled study of cooking skills, eating behaviors, and physical activity of 4th graders and their families. BMC public health. 2016 Dec;16(1):1-5.	A
Delamater AM, Pulgaron ER, Rarback S, Hernandez J, Carrillo A, Christiansen S, Severson HH. Web-based family intervention for overweight children: a pilot study. Childhood obesity. 2013 Feb 1;9(1):57-63.	A

Dickinson WP, Glasgow RE, Fisher L, Dickinson LM, Christensen SM, Estabrooks PA, Miller BF. Use of a website to accomplish health behavior change: if you build it, will they come? And will it work if they do?. The Journal of the American Board of Family Medicine. 2013 Mar 1;26(2):168-76.	B
Farrow C, Belcher E, Coulthard H, Thomas JM, Lumsden J, Hakobyan L, Haycraft E. Using repeated visual exposure, rewards and modelling in a mobile application to increase vegetable acceptance in children. Appetite. 2019 Oct 1;141:104327. doi: 10.1016/j.appet.2019.104327. Epub 2019 Jun 19. PMID: 31228505.	C
Grimes CA, Booth A, Khokhar D, West M, Margerison C, Campbell KJ, Nowson CA. Digital education to limit salt in the home (DELISH) program improves knowledge, self-efficacy, and behaviors among children. Journal of nutrition education and behavior. 2018 Jun 1;50(6):547-54.	A
Carlin A. Can intelligent personal systems help families to be more physically active? Available from: https://doi.org/10.1186/ISRCTN16792534 (Accessed on 15 November 2022)	A
Kato-Lin YC, Kumar UB, Prakash BS, Prakash B, Varadan V, Agnihotri S, Subramanyam N, Krishnatray P, Padman R. Impact of Pediatric Mobile Game Play on Healthy Eating Behaviour: Randomized Controlled Trial. JMIR mHealth and uHealth. 2020;8(11):e15717.	C
Matheson D. Family-Based Nutrition Intervention for Latino Children. Available from: https://www.cochranelibrary.com/central/doi/10.1002/central/CN-01511657/full (Accessed on 15 November 2022)	A
Prout E, 2017. Gamification and Energetic Behavior Changes. Available from: https://ichgcp.net/clinical-trials-registry/NCT03050840 (Accessed on 15 November 2022)	A
Piatkowski C, Faulkner GE, Guhn M, Mâsse LC. User Characteristics and Parenting Practices Associated with Adolescents' Initial Use of a Lifestyle Behavior Modification Intervention. Childhood Obesity. 2020 Sep 1;16(6):367-78.	B
Schoffman DE. *Enhancing Parent-Child Communication and Promoting Physical Activity and Healthy Eating Through Mobile Technology: A Randomized Trial* (Doctoral dissertation, University of South Carolina).	D
Thompson D, Baranowski T, Cullen K, Watson K, Liu Y, Canada A, Bhatt R, Zakeri I. Food, fun, and fitness internet program for girls: pilot evaluation of an e-Health youth obesity prevention program examining predictors of obesity. Preventive medicine. 2008 Nov 1;47(5):494-7.	A
Wong RS, Yu EY, Wong TW, Fung CS, Choi CS, Or CK, Liu KS, Wong CK, Ip P, Lam CL. Development and pilot evaluation of a mobile app on parent-child exercises to improve physical activity and psychosocial outcomes of Hong Kong Chinese children. BMC public health. 2020 Dec;20(1):1-3.	A

Reasons for exclusion; number of studies indicted in parentheses – A: Irrelevant study design/protocol (11); B: Irrelevant study population (2); C: Irrelevant setting (3); D: Thesis/dissertation/conference proceedings (1)

Supplementary Table S3. Interventions available on marketplace (identified from web searching).

Programs	Go4Fun Online (NSW)	Better Health Program Online (WA)	Think, Eat and Move (TEAM) Program Online	Weight Watchers/Kurbo	Nestle Healthy Active Kids
**Creator**	NSW Health and Better Health Company	Better Health Company	Better Health Company; funded by Central and Eastern Sydney PHN	Weight Watchers + Stanford University	Nestle
**Description**	Treatment. Obesity program launched in 2017	Treatment. Similar program to Go4Fun Online but with different branding	Treatment. Appears to be a teen variation of Go4Fun Online / Better Health Program	Treatment. Youth-targeted weight loss service	Prevention. Focuses on teacher-led learning, previously targeted to families
**Target audience**	Children and parents/carers	Children and parents/carers	Teens and parents/carers	Children and teens, and parents	Teachers
**Digital component**	Interactive web-based program	Interactive web-based program	Interactive web-based program	App and weekly coaching service (paid subscription)	Interactive web-based program
**Website**	https://go4funonline.com.au/ (accessed on 2 February 2021)	https://betterhealthprogram.org/ (accessed on 2 February 2021)	https://www.thinkeatandmove.org/ (accessed on 2 February 2021)	https://kurbo.com/ (accessed on 2 February 2021)	https://www.n4hk.com.au/ (accessed on 2 February 2021)

Supplementary Table S4. Characteristics of included studies.

Author (Year) Country	Study aims	Study design and participants	Intervention components and usage	Theoretical framework
Ahmad (2018) Malaysia [17]	To evaluate the effectiveness of social media and face-to-face sessions in a family-based intervention on the primary outcome of body mass index z-score and secondary outcomes of waist circumference percentile and percentage total body fat.	*Study design:* RCT<br>INT (*n*=67): 4 weeks of weekly training for parents to change child behavior and 3 months of weekly booster which consisted of weekly one-hour sessions using a WhatsApp group.<br>CON (*n*=67): Waitlist control<br><br>*Participants:*<br>134 parent-child dyads. Children aged 7-10 years. All participants were Malay females.<br><br>*Attrition rate:* 9%<br>122 of 134 families completed 6-month assessments.	▪ Face-to-face session (week 1 and 4), subsequently uploaded to Facebook<br>▪ Sessions on Facebook Group (week 2 and 3)<br>▪ WhatsApp group for information sharing<br><br>▪ 96.9% of parents participated in WhatsApp and 81.3% in Facebook respectively, compared to 68.8% for session one and 42.2% for session two of the face-to-face sessions.<br>▪ About equal number of replies (e.g., thank you, thumbs up, smiley) were observed in both WhatsApp (75 responses) and Facebook (73 responses).<br>▪ Among 111 responses around sharing of problems or progress, sharing information about food and physical activity, 91% (101 responses) were posted in WhatsApp compared to Facebook (9%, 10 responses).<br>▪ Greater participation in WhatsApp may be due to the longer duration of contact with parents (3 months) in WhatsApp compared to Facebook (1 month).	INT was developed by researchers using Social Cognitive Theory (SCT). The elements of behavior modification skills in the SCT include self-monitoring, goal setting, self-efficacy, problem solving, relapse prevention, and stimulus control. Parents were encouraged to acquire authoritative parenting skills, practice healthy behaviors and improve self-efficacy of child's healthy behaviors.
Bakirci-Taylor (2019) USA [18]	To explore the potential of mHealth using smartphones to improve fruit and vegetable intake in children.	*Study design:* RCT<br>INT (*n*=15): 10 weeks of the mobile Jump2Health intervention which included 3 components: a mobile website (Jump2Health), social media (Facebook page), and short message service or text messages. The Facebook page provided information that was unavailable on the mobile Jump2Health website, but it also mentioned and reinforced information and text found	▪ Website (main content)<br>▪ Facebook page (additional content)<br>▪ Mobile text messages (about FV consumption)<br><br>▪ 64% (*n*=7 of 11) participants registered for website, all have accessed the website through smartphones, 86% visited weekly.	The posts were developed using the constructs of the Social Cognitive Theory to better address parental barriers related to vegetable and fruit consumption. Cooking videos and recipes, strategies to address picky

Author (Year) Country	Study aims	Study design and participants	Intervention components and usage	Theoretical framework
		on the website and promoted linked resources on the website. CON (*n*=15): Control group did not receive access to the website or social media; they received 12 text messages only about physical activity.<br><br>*Participants:*<br>30 parent-child dyads. Children were aged 3-8 years. All parents were female, married, and predominantly Caucasian. Over 70% had at least a bachelor's degree and 40% reported incomes of ≥$75,000.<br><br>*Attrition rate:* 17%<br>25 of 30 families completed week 10 assessments.	▪ All 11 participants reported readily use of Facebook page than website, with 83% engagement rate for Facebook.<br>▪ Parents noted interaction among Facebook participants as an important feature.<br>▪ 72% reported that they preferred 1 text message/week preferably on earlier days of the week: Sunday (32%), Monday (40%), Tuesday (36%).	eating, and food budgeting and meal planning were included to address the behavioral capability construct of Social Cognitive Theory.
Baranowski (2003) USA [19]	To test the *Girls health Enrichment Multisite Studies (GEMS) Fun, Food, and Fitness Project (FFFP)* interventions and associated measurements using a randomized clinical trial design that included a comparison group.	*Study design:* RCT<br>INT (*n*=19): 4-week summer day camp, followed by an 8-week home Internet intervention for the girls and their parents which included weekly behavioral/ environmental foci. The treatment camp blended usual camp activities with activities specially designed for *GEMS-FFFP.*<br>CON (*n*=16): The control camp experienced only the usual camp activities at that site. Control group was asked to log on to website once a month and was provided general health information.<br><br>*Participants:*<br>35 parent-child dyads. Children aged 8 years with a BMI in the 50th percentile for age and gender specific BMI. All participants were female African Americans. Majority had a household income of >$40,000, college graduate or higher education.<br><br>*Attrition rate*: 11%	▪ Summer day camp (4 weeks in duration)<br>▪ Website (new content weekly for 8 weeks)<br>▪ Weekly email and phone reminders to log on<br>▪ Overall website log-on rates were 48% for girls and 47% for parents.<br>▪ User engagement is challenging and suggested that attention should be paid to characteristics of the Web content, incentives, reminders, and characteristics of the participants, and whether these vary for differing age and gender groups.	The INT was underpinned by Social Cognitive Theory and suggested that child behavior was influenced by a triad of preference, availability, and skill.

Author (Year) Country	Study aims	Study design and participants	Intervention components and usage	Theoretical framework
		31 of 35 families completed week 12 assessments.		
Chai (2021) Australia [20]	To investigate the feasibility and preliminary efficacy of a novel 12-week family-focused online telehealth nutrition intervention in improving child weight status and dietary intake.	*Study design:* RCT<br>INT: Both INT 1 (*n*=16) and 2 (*n*=15) groups received two telehealth consultations delivered by a dietitian, 12 weeks access to a nutrition website and a private Facebook group. INT 2 group received additional text messages.<br>CON (*n*=15): Waitlist control<br><br>*Participants:*<br>46 parent-child dyads. Children (mean age 9 years) were predominantly male, have overweight/obesity and resided with both biological parents. Parents (mean age 41 years) were predominantly female, of middle SES, living in major cities, have overweight/obesity, and attained certificate/diploma or postgraduate degree.<br><br>*Attrition rate*: 22%<br>36 of 46 families completed week 12 assessments.	▪ Website (new content weekly for 12 weeks). 77% accessed the website. Highest average number of website visits was on Tuesdays (day of the week) and around 16:00 (time of the day).<br>▪ Telehealth dietitian (session 1: 35 minutes, session 2: 20 minutes). Attendance rates for both sessions were 96% and 78%, respectively.<br>▪ Facebook group. 80% accessed the Facebook group.<br>▪ Mobile text messages (various frequency for 12 weeks) received by 29 parents in INT 2 (*n*=15 families).<br>▪ A common trait observed for the three least clicked links was the mobile text messages content did not provide information about the topic of the link.	Mobile text messages were underpinned by Theoretical Domains Framework and COM-B Model.<br><br>Telehealth consultations were guided by the Coventry, Aberdeen, and London – Refined (CALO-RE) taxonomy of behavior change techniques related to healthy eating.
Cullen (2017) USA [21]	The intervention objectives were to improve parent and child fruit and vegetable intake.	*Study design:* RCT<br>INT (*n*=92): Approximately 8 weeks web-based program for African American families that was designed to promote healthy home food environments, positive parental behaviors related to improving dietary behaviors of family members and goal setting.<br>CON (*n*=34): Control<br><br>*Participants:*<br>126 parent-child dyads. Children aged 8-12 years. All participants were African American. Majority of parents were female, aged <40 years, college graduate or higher education, and have less than two children.	▪ Website (narrated graphic story viewing).<br>▪ Eight stories follow the Johnson family (an African American family with two 8- to 12-year-old children) as they try to develop healthier eating habits.<br>▪ After viewing the weekly story, the parents had a challenge (goal) to complete during the next week and viewed a family food problem. Tip sheets targeting the session content and recipes could be downloaded from the website.<br>▪ The log on rate over the 8-week program was 86% for those who completed Post 2	NR

Author (Year) Country	Study aims	Study design and participants	Intervention components and usage	Theoretical framework
		Attrition rate: 32%<br>86 of 126 families completed 6-month assessments.	measurements; 66% logged onto all the eight sessions. There was no difference in log on rates by condition.	
De Lepeleere (2017) Belgium [22]	The aims were to evaluate the effect of 'Movie Models' on the child's behavior (PA, screen-time, healthy diet) and to investigate the intervention effect on parenting practices and parental self-efficacy.	*Study design:* Quasi-experimental controlled trial<br>INT (*n*=45-49): 4 weeks access to website (health promoting videos); content delivered weekly over four weeks; contact time ~ 2 mins per video (22 videos)<br>CON (*n*=63): Waitlist control<br><br>*Participants:*<br>135 parents of a primary school aged child. Majority of parents were female and from a medium-high SES.<br><br>*Attrition rate*: 53%<br>63 of 135 families completed 4-month assessments.	▪ Website (online videos followed by narrator explanation)<br>▪ 22 short videos (2 minutes each) showing a difficult child-parent scenario followed by an appropriate reaction of the parent, then a narrator explains the parenting practices showed in the video.<br>▪ Each video was watched by 93.3–100% of parents from the intervention group.	Intervention Mapping Protocol was applied in development of the intervention based on Self Determination Theory and Social Cognitive Theory focusing on parental modeling, attitude and self-efficacy related to PA, screentime and dietary behavior.
Jake-Schoffman (2018) USA [23]	To test the feasibility, acceptability, and preliminary effectiveness of two remotely delivered family-based health promotion programs for improvements in parent–child dyad's physical activity and healthy eating.	*Study design:* RCT<br>INT (*n*=16): Dyads were asked to self-monitor using a mobile responsive design website made for the intervention for 12 weeks.<br>CON (*n*=17): Dyads randomized to the Tech program were asked to self-monitor via study-provided paper logs.<br><br>*Participants:*<br>33 parent-child dyads. Majority of parents were female, Caucasian, college graduates, and have obesity. Majority of children were female, Caucasian, aged 11 years, and have a healthy weight.<br><br>*Attrition rate*: 6%<br>31 of 33 families completed week 12 assessments.	▪ Mobile website with messaging function and features such as side-by-side graphs to show the daily progress of parents and children toward study goals, and a messaging feature where parents and children could send messages of support and encouragement to one another to help reinforce behavioral goals.<br>▪ Website included sections directed to parents, separate sections for children, and a section for the family, to encourage collaboration.<br>▪ Email newsletter<br>▪ Study reported high adherence to self-monitoring protocols with parents and children using the mobile website for step and food logs. Study also reported moderately high utilization of program materials.	Intervention materials were informed by the Social Cognitive Theory and the Theory of Planned Behavior and offered overall information about setting small attainable goals, identifying and overcoming obstacles to behavior change, and encouraging a shift in attitudes toward PA and heathy eating in the family unit. The Tech+ intervention group also incorporated elements of Family Systems Theory and

Author (Year) Country	Study aims	Study design and participants	Intervention components and usage	Theoretical framework
				conceptualized parent–child relationships in the context of reciprocal interactions.
Johansson (2020) Sweden [24]	The aim was to study feasibility in terms of trial design, mHealth usage, compliance, and acceptability of the treatment from parents and clinicians.	*Study design:* RCT<br>INT (*n*=15): 6 months daily self-monitoring of weight recorded via a mobile app used by parents, a website in which clinicians could track treatment progress, prespecified treatment goals for change in degree of obesity shown in the app and on the website, and text message interactions between clinicians and parents. In addition to the mHealth approach, the intervention group received standard care (clinical appointment).<br>CON (*n*=13): Control group received standard care only which followed the procedure for obesity treatment at each pediatric clinic.<br><br>*Participants:*<br>28 children aged 5-12 years who have obesity according to the International Obesity Task Force (IOTF) with parents who speak Swedish.<br><br>*Attrition rate*: 11%<br>25 of 28 families completed 6-month assessments.	▪ Daily weighing at home on scales with no displays to indicate weight and data were transferred to the mobile app (for families) and to the clinic's interface (website).<br>▪ The clinicians were instructed to check the participants' weight charts on the clinic's interface at least weekly and give feedback via text messages.<br>▪ A wrist-worn activity monitor was connected to a gamified app which prompt for physical activity to generate rewards that were displayed in the app.<br>▪ The mHealth approach was completed by 12/15 children (completers) regularly throughout the entire study period. All parents reported that they followed their child's physical activity through the gamified app.<br><br>▪ Clinic appointment cancellation was higher in standard care control group (85%) compared to intervention group (40%).	NR
Knowlden (2015) USA [25]	To compare the effects of the *EMPOWER* intervention with an equivalent knowledge-based intervention on the four identified obesogenic protective factors in	*Study design:* RCT<br>INT (*n*=29): 4 weekly audiovisual presentation (30 mins per session) via website and 1 booster session<br>CON (*n*=28): Online healthy lifestyle information<br><br>*Participants:*<br>57 mothers with children aged 4-6 years. Mothers (mean age of 36 years) were predominantly	Each of the five online sessions included a 10- to-15-minute audiovisual presentation, an interactive online worksheet, and a discussion board post designed to increase knowledge supplemented each module.	The INT was designed to reify and improve five Social Cognitive Theory constructs in mothers: environment, emotional coping, expectations, self-control, and self-efficacy.

Author (Year) Country	Study aims	Study design and participants	Intervention components and usage	Theoretical framework
	children ages 4 to 6 years as measured through a valid and reliable behavior log.	Caucasian, married, unemployed/homemakers. Children (mean age of 5 years) were primarily Caucasian, male, with a mean age of 5 years.<br><br>*Attrition rate*: 12%<br>50 of 57 families completed week 8 assessments.		
Maddison (2014) New Zealand [26]	The *SWITCH* (Screen-Time Weight-loss Intervention Targeting Children at Home) study aimed to determine the effect of a home-based, family-delivered intervention to reduce screen-based sedentary behavior on body composition, sedentary behavior, physical activity, and diet over 24 weeks in overweight and obese children.	*Study design:* RCT<br>INT (n=127): Delivered over 20 weeks, consisted of a face-to-face meeting with the parent/caregiver and the child to deliver intervention content; tv monitoring device; monthly newsletters.<br>CON (n=124): Control group was asked to continue with their usual behavior and had access to a generic *SWITCH* public website. They were provided intervention material post follow-up.<br><br>*Participants:*<br>251 parent-child dyads. Children (mean age of 11 years) were predominantly male and of Pacific origin.<br><br>*Attrition rate*: 5%<br>238 of 251 families completed week 24 assessments.	▪ Initial face-to-face session<br>▪ Website (health information and links to community-based activity programs)<br>▪ Time Machine TV monitoring device (2 devices per family)<br>▪ Activity pack for children<br>▪ The Time Machine was connected to a TV, or other media device (e.g., DVD player, video game console), but it was not possible to connect the device to a computer. Each Time Machine came with 30 tokens, with each token allowing 30 minutes of viewing time; however, caregivers were able to allocate these as they chose.<br>▪ Almost half (46%) of participants reported never using the Time Machine to budget their child's television or computer use; however, 57% reported using any of the strategies discussed in the monthly newsletters.	Intervention content was grounded in Social Cognitive Theory and behavioral economics theory. The study focused on implementing changes to the home and family environment, as well as providing suggestions for parents to make personal and behavioral changes.
Morgan (2019) Australia [27]	To evaluate a novel program designed to improve the physical activity levels of fathers and their daughters.<br><br>To examine the impact of the	*Study design:* RCT<br>INT (n=74): 90-minute group sessions weekly for 8 weeks that included educational and practical components. They were provided with a web-based app at the conclusion of the program for long-term maintenance.<br>CON (n=79): Waitlist control<br><br>*Participants:*	▪ 8 weekly group sessions (90mins)<br>▪ Printed resources<br>▪ App: To encourage long-term behavior maintenance, families were provided with access to a web-based app at the conclusion of the program, which included a variety of fun physical activities for daughters and fathers to	The program targeted the core constructs of Self-Determination Theory (i.e., autonomy, competence, relatedness) and Social Cognitive Theory.

Author (Year) Country	Study aims	Study design and participants	Intervention components and usage	Theoretical framework
	program on (i) daughters' fundamental movement skills competency, (ii) fathers' and daughters' screen-time, and (iii) fathers' physical activity parenting practices.	153 father-child dyads. Children were aged 4-12 years. Most fathers were employed, born in Australia, and were married or living with a partner (99%). Families were represented from most socio-economic areas.<br><br>*Attrition rate*: 18%<br>125 of 153 families completed 9-month assessments.	complete and track together weekly.<br>▪ The app was used at least once by 65 (83%) intervention group families. Fathers and daughters completed a median of 13 (IQR: 3, 24; range: 0–53) app activities across the intervention period.	
Perdew (2021) Canada [28]	To evaluate the effectiveness of a 10-week blended family-based childhood obesity management program, relative to a wait-list control, in improving child body mass index (BMI) z-scores, child lifestyle behaviors, parental support for healthy eating and physical activity, and self-regulation for healthy eating and physical activity support.	*Study design:* Quasi-experimental design<br>INT (*n*=48): The program provided 10 weekly face-to-face 90-minute sessions, four community-based activities (i.e., family grocery store tour), and the interactive web-portal.<br>CON (*n*=23): Waitlist control<br><br>*Participants:*<br>71 parent-child dyads. Children were aged 8-12 years; at or above 85th percentile for BMI for age and sex.<br><br>*Attrition rate*: 13%<br>62 of 71 families completed week 10 assessments.	▪ 10 weekly face-to-face 90-minute sessions,<br>▪ Four community-based activities (i.e., family grocery store tour), and<br>▪ Following the in-person sessions, 10 weekly online interactive lessons were made available to the families using a web portal.<br>▪ The weekly online lessons complemented the in-person sessions by offering additional resources about healthy living, weekly physical activity challenges, family recipes ideas, personal diaries for family goal setting and monitoring, and an online discussion forum.<br>▪ Attendance of weekly in-person sessions was 58%.<br>▪ The mean online portal engagement for both groups for log-in frequency was 3.29 times, mean weekly portal engagement minutes was 14.57 minutes, and families accessed on average 22.19% of the online portal content.	The INT development was guided theoretically by the multi-process action control framework which introduces seven constructs that are precursors of health behaviors (attitude, motivation, self-efficacy, opportunity, behavioral regulation, identity and habit).
Rangelov (2018)	To examine the effect of a Social	*Study design:* RCT<br>INT: 8 weeks access to website (parents) and a personalized and	▪ Website (weekly nutrition content and a forum for	The Social Marketing framework was

Author (Year) Country	Study aims	Study design and participants	Intervention components and usage	Theoretical framework
Switzerland [29]	Marketing healthy nutrition program on children's food intake. It was aimed to assess if additional support parents received through e-mail or mobile text messages resulted in additional behavior change of their child over that of the Web-only group.	tailored letter by post (children). The emails (INT 1, *n*=196) and mobile text messages (INT 2, *n*=194) were used as weekly reminders to prompt parents to visit the Website. The email also provided a short summary of the weekly theme.<br>CON (*n*=218): Website only<br><br>*Participants:*<br>608 parent-child dyads. Children (mean age of 8.5 years) were in the first two years of secondary school and about equal proportion of boys and girls.<br><br>*Attrition rate*: No participant withdrew from the program, but only 608 of 735 children (452 parents) had complete data.	participants discussion together and with a dietitian)<br>▪ Emails and mobile text messages were sent as weekly reminders to prompt parents to visit the website.<br>▪ Overall, 39% to 46% participants have visited the website.	used in the development of the intervention, incorporating all six elements of the marketing mix: product, place, price, promotion, policy and partnership.
Thompson (2015) USA [30]	To present the outcome evaluation of an online serious videogame promoting FV to children that systematically varied action and coping implementation intentions and examined the short- and longer-term effects on FV intake.	*Study design:* RCT<br>All groups played the 10-episode (1-hour each) online videogame. The groups varied only on type of implementation intention created after setting a goal to eat FV:<br>*Action* (*n*=97): Children set a FV goal and then created an action plan specifying how they would meet the goal.<br>*Coping* (*n*=98): Children set a goal to eat more FV; they then created a coping plan that identified a potential barrier that might keep them from meeting their goal.<br>*Both* (*n*=95): Children set a goal to eat FV, then created both action and coping plans by first creating an action plan, followed by the selection of a barrier and solution as described above.<br>*None* (*n*=97): Children played the game, but only set a goal to eat FV (i.e., they did not create an action or coping implementation intention).	▪ 10 episodes of online video game<br>▪ 10 newsletters (for parents)<br>▪ 10 installment parents only website (The parent intervention was connected to the child intervention.)<br>▪ Child participation rate was high: 91% of children played all 10 episodes of the videogame.<br>▪ Parents' participation rates varied; with 28% reported reading more than 6 newsletters. 60% visited website at least 6 times.	Multiple theories were used to guide various aspects of behavior change: Social Cognitive Theory; Self Determination Theory; Behavioral Inoculation Theory; Maintenance Theory; and the Elaboration Likelihood Model

Author (Year) Country	Study aims	Study design and participants	Intervention components and usage	Theoretical framework
		Participants:<br>400 parent-child dyads. Children were in 4th or 5th grade (around 9-11 years). Almost evenly distributed by gender (female, 52.7%) and were of diverse ethnicity (White-36.8%, Hispanic 27.4%, African American 26.4%). Parents were mostly female (96.3%), White (40.3%), married (77.5%), and 40–59 years old (55.3%). Highest level of household education was predominately post-graduate study (36.7%), and average household income was > $61,000 (57.6%).<br><br>*Attrition rate*: 3%<br>387 of 400 families completed 6-month assessments.		
Trost (2021) Australia [31]	To evaluate the effectiveness of the Moovosity™ programme, a novel digital application to increase FMS proficiency in 3- to 6-year-old children.	*Study design:* RCT<br>INT (*n*=17): Families were free to use the app ad libitum but were asked to complete a minimum of three Moovosity™ activities per week, over 8 weeks. Parents were sent mobile text messages and emails every two-weeks.<br>CON (*n*=17): Waitlist control<br><br>*Participants:*<br>34 parent-child dyads. Children aged 3-6 years and predominantly were Caucasian. Parent were college graduate or higher education.<br><br>*Attrition rate*: None<br>All 34 families completed week 8 assessments.	▪ 8-week access to Moovosity™ mobile application (app) designed to promote the development of FMS and increase physical activity in young children<br>▪ Families were free to use the app ad libitum but were asked to complete a minimum of three Moovosity™ activities per week.<br>▪ Mobile text messages and Emails fortnightly reminders for use of the app<br>▪ 70.6% families used the app at least 2 times per week, including 35.3% who used 3 or more times a week.	NR
Trost (2014) USA [32]	To evaluate the effects of active video gaming on physical activity and weight loss in children participating in	*Study design:* RCT<br>All participants received a comprehensive family-based pediatric weight management program (*JOIN for ME*).<br>INT (*n*=34): Participants in the program and active gaming group received hardware consisting of a	▪ Video games using a game console and motion capture device with 2 active sports games (at second treatment and week 9).<br>▪ INT program is 16 weekly group sessions.	The use of a theoretical framework was not reported; however, intervention strategies have included goal setting,

**Author (Year) Country**	**Study aims**	**Study design and participants**	**Intervention components and usage**	**Theoretical framework**
	an evidence-based weight management program delivered in the community.	game console and motion capture device and 1 active game at their second treatment session and a second game in week 9 of the program.<br>CON (*n*=41): Participants in the program-only group were given the hardware and 2 games at the completion of the 16-week program.<br><br>*Participants:*<br>75 parent-child dyads. Children aged 8-12 years, had a BMI greater than the 85th percentile for sex and age and predominantly were female and White. Parents were college graduate or postgraduate.<br><br>*Attrition rate*: 20%<br>60 of 75 families completed week 16 assessments.	▪ Retention rates at 8 and 16 weeks were 32 of 34 (94%) and 26of 34 (76%), respectively, for the P + AG group, and 40 of 41(98%) and 34 of 41 (83%), respectively, for the PO group.	reinforcement, modeling, and changing the home environment.
Wald (2018) USA [33]	To assess the feasibility of an intervention combining 6 face-to-face group counselling sessions with a 1-year longitudinal web-based component to help parents of overweight children promote healthy behavior change.	*Study design:* RCT<br>INT (*n*=38): The intervention was composed of 6-in-person group sessions and a customized website over 12 months.<br>CON (*n*=-35): Received standard care but no access to website.<br><br>*Participants:*<br>73 parent-child dyads. Children (mean age 5 years) were predominantly non-Hispanic and had overweight/obesity. Mothers were predominantly aged <40 years, college graduate or higher education, and married.<br><br>*Attrition rate*: 59%<br>30 of 73 families completed 12-month assessments.	▪ 6 weekly group counseling sessions<br>▪ 1-year access to Website with weekly update including health topics related to nutrition and physical activity, local resources for current activities for children and families, personal stories that emphasized authoritative parenting, interactive discussion group, and Ask the Expert.<br>▪ Parents were encouraged to share their triumphs and challenges so that all might benefit.<br>▪ 38% completed at least 4 of the 6 sessions<br>▪ All 24 parents accessed the website at least once during the initial 6 weeks, 88% accessed more than once. Only 29% accessed website after the 6 weeks face-to-face sessions.	INT is based on Self-Determination Theory, which holds that three basic psychological needs must be satisfied to foster well-being: autonomy, competency, and relatedness.
Williamson (2005)	A randomized controlled trial	*Study design:* RCT	▪ 4 face-to-face counselling sessions during the first 12 weeks	The use of a theoretical

Author (Year) Country	Study aims	Study design and participants	Intervention components and usage	Theoretical framework
USA [34]	tested the efficacy of an internet-based lifestyle behavior modification program for African American girls over a 2-year period of intervention.	INT (*n*=28): Interactive website and 4 face-to-face sessions of behavior modification over 12 weeks focused on goal setting, behavioral contracting, monitoring of progress, and problem solving. Participant-initiated weekly emails with counsellor.<br>CON (*n*=29): General website and 4 face-to-face sessions of nutrition education from a registered dietitian but behavioral changes for the adolescent or parent were not prescribed, and internet counselling was not provided.<br><br>*Participants:*<br>57 African American girls aged 11-15 years.<br><br>*Attrition rate*: 30%<br>40 of 57 families completed 24-month assessments.	of program. The intervention used a family-oriented format, i.e., a program that invited the parents, the child, and other members of the family to be involved using mutual problem-solving and behavioral contracting.<br>▪ Interactive website components included interactive graph to track exercise, interactive food monitoring worksheets with instant feedback on food choices, problem solving worksheet (online), and a quiz followed every weekly lesson with instant feedback provided.<br>▪ Online counseling included weekly email communication with counsellor for feedback on program components (e.g., quizzes, lessons, weight graphs, goal setting, clinic appointments).<br>▪ The mean total "hits" on the interactive website by adolescents and parents was significantly higher (2 times greater) than control website which was non-interactive and provide health education information only.	framework was not reported; however, intervention strategies have included problem solving, goal setting, and behavioral contracting.

RCT: Randomized Controlled Trial; INT: intervention group; CON: control or comparison group; NR: not reported; FV: fruit and vegetables.

Supplementary Table S5. Intervention effects on child outcomes.

Author (Year) Country	Overall findings*	Anthropometry*	Dietary intake*	Physical activity*	Sedentary, Screen time, Sleep*
Ahmad (2018) Malaysia [17]	BMI z-scores were significantly reduced in the intervention group compared to the wait-list group for all the children at 6-month post-training. For waist circumference percentile and body fat percentage, the intervention group experienced a significant reduction compared to the wait-list group, within the obese subgroup and within the overweight subgroup.	Decreased zBMI (value NR) Decreased waist circumference percentile (value NR) Decreased body fat percentage (value NR)	N/A	N/A	N/A
Bakirci-Taylor (2019) USA [18]	Skin carotenoids of both children and parents showed significant Week x Treatment interactions in the INT group compared with CON (*p*<0.001) indicating increased veg intake.	NS changes in BMI percentiles	Increased variety of veg consumed per day (+0.69; *p*=0.02)	N/A	N/A
Baranowski (2003) USA [19]	There were no significant changes.	NS changes in BMI and waist circumference (cm)	NS changes in FJV (serves), total kcal consumed and SSB (serves) intake	NS changes in accelerometer (24 hours) counts/min	N/A
Chai (2021) Australia [20]	Percentage energy from EDNP food was reduced and percentage energy from nutrient-rich core food was increased in *Telehealth+SMS* when compared to CON.	NS changes in BMI, BMI z-scores and waist circumference (cm) at week 12	Decreased overall energy intake (-2835kJ/d; *p*=0.027; *Telehealth* vs CON) Decreased EDNP food intake (-11%E; *p*=0.049; *Telehealth+SMS* vs CON) Increased core food intake (+10.8%E; *p*=0.045; *Telehealth+SMS* vs CON)	NS changes in PA level scores	N/A
Cullen (2017) USA [21]	Home availability of juice (*p*<0.05), vegetables (*p*<0.01), and low-fat/fat-free foods (*p*<0.05) were significantly higher in INT at 2-month. Parent menu planning skills	N/A	NS changes in FJV and SSB intake (frequency/week)	N/A	N/A

Author (Year) Country	Overall findings*	Anthropometry*	Dietary intake*	Physical activity*	Sedentary, Screen time, Sleep*
	were significantly higher in INT at 6-month. Both INT and CON groups showed significant increases for home juice/fruit availability, parent modelling, food preparation practices, and menu planning, and a significant decrease in home sugar sweetened beverage availability (all $p<0.05$).				
De Lepeleere (2017) Belgium [22]	Most significant intervention effects were found for more complex parenting practices (e.g., an increase in motivating the child to eat fruit). Subgroup analyses showed that the intervention had more effect on the actual parenting practices related to PA, screen-time and healthy diet in parents of older children (10–12 years old), whereas intervention effects on parental self-efficacy related to those behaviors were stronger in parents of younger children (6–9 years). Unexpectedly, parents of INT group had a significant decrease in their 'self-efficacy concerning having vegetables available' ($p=0.03$) after 4 months.	N/A	NS changes in FV and soft drinks intake (portions/week)	NS changes in minutes of active transportation	NS changes in minutes of screen time
Jake-Schoffman (2018) USA [23]	There were no significant Group x Time x Parent or Group x Time effects on any of the intervention outcomes: minutes of MVPA (accelerometer), daily steps (pedometer), servings of fruit, vegetables, fast food, and SSBs.	N/A	NS changes in FV, fast food and soft drinks intake (servings/week)	NS changes in MVPA (minutes)	N/A
Johansson (2020)	At 6 months the intervention group had a greater	Reduced zBMI; $p=0.017$ at 3 months; $p=0.002$ at 6 months;	N/A	N/A	N/A

Author (Year) Country	Overall findings*	Anthropometry*	Dietary intake*	Physical activity*	Sedentary, Screen time, Sleep*
Sweden [24]	reduction in standardized BMI than standard care.	*Provement* vs control group			
Knowlden (2015) USA [25]	The EMPOWER arm of the trial resulted in an overall increase of 1.680 daily cups of fruits and vegetables consumed by children, relative to the comparison group (p<0.001, 95% confidence interval. Web-based maternal-facilitated interventions can induce sustained effects on child behaviors.	N/A	Increased FV intake at posttest (1.613, p<0.001) and follow-up (1.68; p<0.001) Increased sugar-free beverage intake at posttest (1.07; p<0.001) and follow-up (0.093; p=0.772)	Increased PA level at posttest (32.66; p=0.006) and follow-up (13.028; p=0.313)	Decreased screen-time at posttest (-39; p=0.019) and at follow-up (-5.545; p=0.503)
Maddison (2014) New Zealand [26]	There was no significant difference in change of BMI z-scores between the intervention and control groups, although a favorable trend was observed (−0.016; 95% CI: −0.084, 0.051; p=0.64). There were also no significant differences on secondary outcomes, except for a trend towards increased children's moderate intensity physical activity in the intervention group (24.3 min/d; 95% CI: −0.94, 49.51; p=0.06).	NS changes in BMI, BMI z-scores and waist circumference	N/A	NS changes in MVPA (minutes/day)	NS changes in total sedentary time, screen-time and time sleeping (minutes/day)
Morgan (2019) Australia [27]	ITT analyses revealed favorable group-by-time effects for physical activity in daughters (p=0.02, d = 0.4) and fathers (p<0.001, d = 0.7) at 9 months. At postintervention and follow-up, significant effects (p<0.05) were also identified for daughters' fundamental movement skills competence (objective: d = 1.1–1.2; perceived: d = 0.4–0.6), a range of fathers' physical activity parenting practices (d = 0.3–0.8), and screen-time for daughters (d = 0.5–0.8) and fathers (d = 0.4–0.6,	NS changes in BMI z-scores	N/A	Increased step count at 2 months (875; p=0.02) and at 9 months (907; p=0.03) Increased adjusted step count at 2 months (2013; p<0.001) and at 9 months (1583; p<0.001) Increased Test of Gross Motor Development (TGMD) score at 2 months (7.8; p<0.001) and at 9	Reduction in weekday screen time at 2 months (-31; p<0.001) and at 9 months (-30; p<0.001) Reduction in weekend screen time at 2 months (-32; p=0.02) and at 9 months (-38; p<0.03)

Author (Year) Country	Overall findings*	Anthropometry*	Dietary intake*	Physical activity*	Sedentary, Screen time, Sleep*
	postintervention only). Program satisfaction and attendance were very high.			months (6.4; *p*<0.001)	
Perdew (2021) Canada [28]	Children's BMI z-scores were not significantly changed. Intervention group significantly improved their days of moderate-to-vigorous physical activity relative to control; however, child dietary behaviors were not significantly changed. Relative to control, intervention group showed significant improvements in physical activity.	NS changes in BMI z-scores	NS changes in fruit intake, vegetable intake, SSB intake (times per day in a typical week)	Increased MVPA; *p*-value NR	NS changes in sedentary time and screen-time (hours per day)
Rangelov (2018) Switzerland [29]	Overall, the intervention effects were not significantly different across groups. Children increased their daily consumption of fruit and decreased that of sweets regardless of the group they were assigned.	BMI was NR	NS changes in fruit, sweets, SSB intake and water intakes (frequency of consumption). Increased vegetables intake (0.12; *p*<0.05); *Website* + *SMS* vs control	N/A	N/A
Thompson (2015) USA [30]	A significant group-by-time interaction for FV intake (*p*<0.001) was found in only the *Action* group, which had significant increases in FV intake at post 1 (*p*<0.0001) and post 2 (*p*<0.0001). No other significant interactions were observed.	N/A	Increased FV intake at 3 months (0.72; *p*<0.0001) and at 6 months (0.68; *p*<0.0001) in *Action* group. Increased FV intake at 3 months only (0.48; *p*<0.001) in *Coping* group.	N/A	N/A
Trost (2021) Australia [31]	There were no significant intervention effects observed for child PA.	BMI changes were NR	N/A	Improved object control (6, *p*=0.0025); *Moovosity*™ vs control NS changes to locomotor skills (performance score) and PA level (activity index)	N/A

Author (Year) Country	Overall findings*	Anthropometry*	Dietary intake*	Physical activity*	Sedentary, Screen time, Sleep*
Trost (2014) USA [32]	Participants in the program and active gaming group exhibited significant increases in MVPA at week 16 ($p<0.05$). In the program-only group, a decline or no change was observed in the moderate-to-vigorous and vigorous physical activity. Participants in both groups exhibited significant reductions in percentage overweight and BMI z scores at week 16. However, the program and active gaming group exhibited significantly greater reductions in percentage overweight.	Decreased weight (5.5; $p<0.05$)<br><br>Decreased BMI z-scores (0.12; $p<0.05$)	N/A	Increased vigorous PA (2; $p<0.05$)<br>Increased MVPA (5.9; $p<0.05$)	N/A
Wald (2018) USA [33]	Among children with 12-month visits, BMI z-scores decreased from baseline to 12 months in both the control and intervention arms however the mean reductions were not significantly different between the control and intervention groups ($p=0.7492$). The percent of children who reduced their screen time by ≥15% did not differ significantly between the intervention and control groups.	NS changes in BMI z-scores, BMI and BMI%	N/A	N/A	NS changes in screen-time (hours per week)
Williamson (2005) USA [34]	Participants in the intervention group lost significantly ($p<0.05$) more body fat (−1.12±0.47 SE) than the control group 0.43±0.47 SE).<br>There was a significant difference in BMI change between groups (intervention −0.19±0.24 SE, $p<0.05$, control +0.65±0.23 SE, $p<0.05$).<br>Participants in the intervention group significantly reduced fat intake compared with control	NS changes in weight and BMI	NS changes in total energy intake (total kilojoule)	N/A	N/A

Author (Year) Country	Overall findings*	Anthropometry*	Dietary intake*	Physical activity*	Sedentary, Screen time, Sleep*
	group (FFQ) (−145.67±37.67 SE, $p<0.05$)				

BMI: Body Mass Index; N/A: not applicable; NR: not reported; INT: intervention group; CON: control or comparison group; NS: not significant; FJV: fruit, juice and vegetables; PA: physical activity; EDNP: energy dense nutrient poor; SSB: sugar sweetened beverages; FV: fruit and vegetables; MVPA: moderate to vigorous physical activity; ITT: intention-to-treat; SE: standard error. *Between-group difference.
